# Joint Bayesian Nowcasting of Severe Acute Respiratory Illness and COVID‐19 Positives in Brazil

**DOI:** 10.1002/sim.70529

**Published:** 2026-04-17

**Authors:** Alba Halliday, Oliver Stoner, Theo Economou, Leonardo Soares Bastos

**Affiliations:** ^1^ MRC Centre for Global Infectious Disease Analysis Imperial College London London UK; ^2^ School of Mathematics and Statistics University of Glasgow Glasgow UK; ^3^ Mathematics and Statistics University of Exeter Exeter UK; ^4^ Climate and Atmosphere Research Center The Cyprus Institute Nicosia Cyprus; ^5^ Instituto de Matemática Pura e Aplicada e Tecnologia IMPATech Rio de Janeiro Brazil; ^6^ Programa de Computação Científica Fundação Oswaldo Cruz Rio de Janeiro Brazil

**Keywords:** Bayesian nowcasting, COVID‐19, infectious disease surveillance, probabilistic forecasting, reporting delays, severe acute respiratory illness (SARI)

## Abstract

Surveillance of severe acute respiratory illness (SARI) in Brazil provides an early warning system for respiratory outbreaks, including COVID‐19, and statistical nowcasting methods are vital for correcting long and variable reporting delays that would otherwise hinder timely decision‐making. Coherent joint prediction of SARI and COVID‐positive SARI in Brazil could improve outbreak response planning and hospital resource management, but most existing nowcasting methods only target one outcome at a time. Furthermore, existing approaches usually focus solely on reconstructing recent incidence rather than forecasting future trends, which could support more proactive risk mitigation. Here, we propose a Bayesian hierarchical framework for joint nowcasting and short‐term forecasting of two outcomes, where one is a strict subset of the other. Building on the generalized‐Dirichlet‐multinomial (GDM) method for correcting delayed reporting, a new beta‐binomial component links SARI and COVID‐positive counts, while separate conditional GDM components capture their distinct delay patterns. To allow for changes over time in the level of disease and delay distributions, flexible latent effects are included in all model components. Using national surveillance data from 2021 to 2024, we conduct a 20‐date rolling prediction experiment across Brazil's 27 federative units. Compared to a well‐established Bayesian nowcasting approach, our joint model achieves about one‐third lower mean absolute error and continuous ranked probability score for contemporaneous nowcasts, with the largest gains in high‐incidence regions. Meanwhile, energy scores indicate improved calibration for joint forecasts of total and COVID‐positive SARI relative to comparable independent models.

**Abbreviations**
COVID: coronavirus disease 2019
CRPS: continuous ranked probability score
GDM: generalized‐Dirichlet‐multinomial
MAE: mean absolute error
MCMC: Markov chain Monte Carlo
NB: negative‐binomial
NobBS: nowcasting by Bayesian smoothing
PSRF: potential scale reduction factor
RMSE: root mean square error
SARI: severe acute respiratory illness
SARS: severe acute respiratory syndrome

## 1 Introduction

Disease surveillance systems are crucial for preventing and responding to infectious disease outbreaks, helping to reduce loss of life and conserve public health resources. Timely information regarding disease prevalence is key, but reporting delays–the interval between an event and its availability in official data–cause observed counts to lag behind reality, potentially leading to late or disproportionate responses.

Delays are inevitable in almost every public health surveillance setting, but their impact depends on their magnitude and variability in relation to how quickly a disease evolves. For example, delays in reporting COVID‐19 hospital deaths in England were usually only a few days [1], yet they still posed a serious challenge in the context of a fast‐changing epidemic.

**FIGURE 1:**
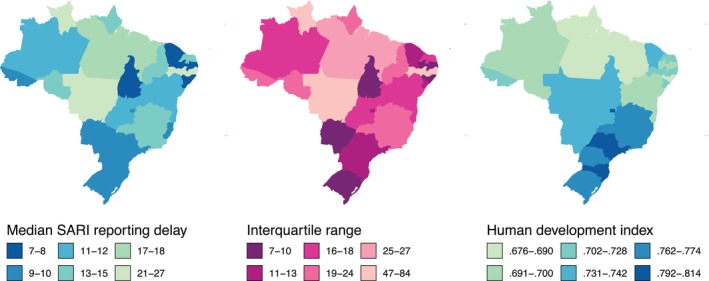
Median severe acute respiratory illness (SARI) reporting delay (days) in 2021–2024 (as defined in Section 3.1), SARI reporting delay interquartile range, and 2021 municipal human development index [2] by federative unit of Brazil.

In Brazil, severe acute respiratory illness (SARI) is a notifiable condition that requires ongoing surveillance. Here, a SARI case is defined as a hospitalized individual with cough or shortness of breath, and with symptom onset in the previous 10 days. Monitoring SARI provides early warning for severe respiratory illness more broadly, capturing cases of COVID‐19, influenza, respiratory syncytial virus, and infections caused by other pathogens. Reporting of SARI can be delayed for weeks, owing to the time between symptom onset, hospitalization, form completion, and entry into the electronic system. Figure 1 shows the median and interquartile range of reporting delays across Brazil's federative units in 2021–2024, highlighting substantial heterogeneity within and between regions. Delays are longest and most variable in more remote areas, such as the Amazon basin, and shorter in more densely populated areas in the east. Delays also appear negatively correlated with the municipal human development index (Figure 1), reflecting differences in healthcare access and administrative infrastructure.

The number of SARI patients testing positive for COVID‐19 is a critical indicator of the burden of COVID‐19 on hospitals. Together, total SARI and the COVID‐positive subset inform both healthcare planning and outbreak response, distinguishing between wider respiratory disease trends and those specific to COVID‐19. However, positive test results are themselves subject to additional reporting delays, since laboratory confirmation and patient record updates take time.

The central statistical challenge, known as “nowcasting,” is to correct for reporting delays and estimate the number of cases that have occurred but are not yet fully reported. Statistical and machine learning approaches aim to do this by learning delay distributions alongside the underlying incidence curve, providing a more accurate and timely picture than raw data alone. Forecasting future incidence–while a natural extension with high practical value–has rarely been attempted in the nowcasting literature, as it requires both adjusting for reporting delays and projecting trends forward in a calibrated way.

For SARI in Brazil, the Oswaldo Cruz Foundation has developed an operational nowcasting system, InfoGripe [3], based on the method of Bastos et al. [4]. This system nowcasts total SARI cases and also reports estimates of the share that tested positive for different pathogens, including SARS‐CoV‐2 (COVID‐19), influenza, respiratory syncytial virus, and rhinovirus. Separately, independent Brazilian scientists have also carried out surveillance of SARI and COVID‐positive SARI up to March 2023 [5], using the method of McGough et al. [6].

COVID‐positive SARI is a strict subset of total SARI, and the two quantities are naturally dependent. Pooling information could therefore improve prediction accuracy and precision–for example, if one quantity is reported more quickly, it may provide predictive information for the other. Existing systems, however, model them independently, reflecting the fact that most established methods for correcting reporting delays are designed for a single outcome. To our knowledge, there is no established framework for joint nowcasting of two outcomes with a strict nesting structure, such as SARI and COVID‐positive SARI.

In this article, we develop a joint modeling approach for SARI and COVID‐positive SARI in Brazil that links the two outcomes hierarchically, while allowing for differences in their reporting delay distributions. Building on existing nowcasting work, we aim to produce short‐term forecasts (up to 4 weeks ahead), extending situational awareness from nowcasting into early warning through forecasting. We apply the model to federative‐unit level data in a rolling prediction experiment, comparing performance against a mainstream nowcasting approach [6] and against an equivalent model without dependence between SARI and COVID‐positive SARI. Although we do not treat this as a spatial problem, independent application across 27 federative units–each with distinct structures and delay patterns–provides a broad basis for comparing performance.

## 2 Background

We begin by introducing some notation to describe the general structure of count data subject to delayed reporting. For cohesion, we will use our own consistent set of labels and indices when referencing existing Bayesian nowcasting methods.

First, we label the total observable count (of disease cases) occurring within time period t (e.g., week) and region s as the “total counts” and denote these as $y_{t,s}$. These totals can be broken down into partial counts, $z_{t,d,s}$, reported at different delay intervals d–for example, d could be the number of weeks since occurrence, with d=0 corresponding to cases reported in the same week as occurrence. The total counts are thus the sum of the partial counts over all possible delays, $y_{t,s}=\sum_{d=0}^{D_{max}}z_{t,d,s}$, where $D_{max}$ is the assumed maximum delay. Together, the mean, variance, and covariance of the partial counts $z_{t,d,s}$ can be thought of as properties of the “delay distribution.” In our data from Brazil, $y_{t,s}$ is the number of SARI cases occurring in week t and federative unit s. Since this section discusses methods that overwhelmingly focus on nowcasting a single disease/outcome or set of counts; we will return to the issue of joint modeling of SARI and COVID‐positive SARI later, in Section 3.

Stoner et al. [1] highlight that the delay distribution may be subject to systematic variation over time and space due to changes in reporting efficiency and resources. Bastos et al. [4] note that reporting delays in Brazil can be greatly impacted by fluctuations in hospital staff absence and workload over the course of an outbreak. Conversely, interventions and awareness associated with the progression of an infectious disease could motivate targeted improvements to local reporting procedures. The distribution of the total counts $y_{t,s}$ will also consist of both systematic and random variability, each of which need to be accounted for. For instance, random variability in the prevalence of the disease could occur between spatial regions due to differing population structures and environments. Incidence of the disease will also vary systematically over time due to the natural spread or mutation of the disease, interventions, and possible seasonal effects.

Capturing all these sources of variability in the data is vital for optimal nowcasting performance but requires models with appropriate flexibility [1]. In Section 2.1, we review existing methods for correcting delayed reporting that aim to achieve this. We focus on approaches within the Bayesian framework to allow for predictive distributions for $y_{t,s}$ given all available data. The quantification of predictive uncertainty is equally important to point prediction accuracy in the context of infectious disease surveillance, so that the risk can be mitigated optimally by considering the full spectrum of plausible outcomes.

### 2.1 Established Methods for Correcting Delayed Reporting

Existing approaches using Bayesian modeling for delay correction in count data can be broadly summarized into two main groups: (1) “conditional models” and (2) “marginal models.”

Conditional models assume a probability distribution $p(z_{t,s}|y_{t,s})$ for the partial counts $z_{t,s}$ that depends on the total counts $y_{t,s}$, which are either seen as historical data (where enough time has passed for those counts to be fully reported) or treated as an unknown quantity to be predicted. The total counts are modeled at a latent level in the hierarchy through $p(y_{t,s})$ (e.g., $y_{t,s}∼\text{Poisson}(\lambda_{t,s})$). In essence, the latent model $p(y_{t,s})$ captures the incidence of the disease, such that the conditional model $p(z_{t,s}|y_{t,s})$ only needs to capture the reporting delay distribution (the delay structures), under the implicit constraint that the partial counts sum up to the total. A key feature of conditional models is that they can ultimately provide an explicit predictive distribution for any unseen total counts/cases given all available data, including historical observed $y_{t,s}$ and more recent partial counts $z_{t,d,s}$. In our view, the current state‐of‐the‐art conditional approach is the generalized‐Dirichlet‐multinomial (GDM) method, first introduced in [7], which we detail in the next subsection.

Most other existing methods can be labeled “marginal” and can be interpreted as probability models for the marginal distribution of $z_{t,d,s}$ after integrating out the total counts, that is, $p(z_{t,d,s})=\sum_{y_{t,s}}p(z_{t,d,s}|y_{t,s})p(y_{t,s})$. In practice, though, $y_{t,s}$ is ignored and only $z_{t,d,s}$ is modeled explicitly. These models typically include features/effects designed to capture the delay distribution and systematic variability in the total counts $y_{t,s}$–while never actually “seeing” the total counts $y_{t,s}$ directly, even though the totals are the predictand of interest.

A well‐established example, Bastos et al. [4], aims to capture spatio‐temporal structure in the total counts and the delay mechanism, as well as optional seasonal effects, through various flexible functions in the mean of $z_{t,d,s}$ in a Bayesian negative‐binomial model:

(1) $$\begin{array}{ll} z_{t,d,s}|\lambda_{t,d,s},\theta_{s} & ∼\text{Negative‐Binomial}(\lambda_{t,d,s},\theta_{s}), \end{array}$$

(2) $$\begin{array}{ll} \log(\lambda_{t,d,s}) & =\mu +\alpha_{t}+\beta_{d}+\gamma_{t,d}+\delta_{d,s}+\Psi_{s}. \end{array}$$

Here, μ is a global intercept, $\alpha_{t}$ is a first‐order random walk capturing the average trend in disease incidence across regions, $\beta_{d}$ and $\delta_{d,s}$ are random walks over delay, $\gamma_{t,d}$ are independent random walks over time for each delay that capture changes in the delay distribution, and $\Psi_{s}$ is composed of a structured and an unstructured spatial effect.

Another widely cited approach within this group is “nowcasting by Bayesian smoothing” (NobBS), proposed by [6]. Like in [4], the NobBS approach assumes a Poisson or negative‐binomial model for $z_{t,d,s}$:

(3) $$\begin{array}{ll} z_{t,d,s}|\lambda_{t,d,s},\theta_{s} & ∼\text{Negative‐Binomial}(\lambda_{t,d,s},\theta_{s}), \end{array}$$

(4) $$\begin{array}{ll} \log(\lambda_{t,d,s}) & =\alpha_{t,s}+\log(\beta_{d,s}), \end{array}$$

(5) $$\begin{array}{ll} \alpha_{t,s}|\alpha_{t-1,s},\sigma_{s}^{\left(\alpha \right)} & ∼\text{Normal}(\alpha_{t-1,s},{\left(\sigma_{s}^{\left(\alpha \right)}\right)}^{2}), \end{array}$$

(6) $$\begin{array}{ll} \beta_{s} & ∼\text{Dirichlet}(p). \end{array}$$

Here, $\alpha_{t,s}$ is a random walk over time that implicitly captures the expected total disease incidence and $\beta_{s}=\beta_{0,s},\ldots ,\beta_{D_{max},s}$ captures the average distribution of cases over delays (the proportion of $y_{t,s}$ reported at each delay). The method proposed by [6] is highly accessible to prospective users via the NobBS package for R [8]. Using the NobBS package, it is possible to run the above model and obtain posterior predictive nowcasting summaries and samples with a single line of code where, if using default settings, the user only needs to supply case list data and specify the column names for the onset date and reporting date.

The main advantage of marginal models–like those proposed by [4] and [6]–is avoiding a hierarchical structure consisting of $y_{t,s}$ and $z_{t,s}|y_{t,s}$. This enables implementation that may be faster for time‐sensitive applications, since inference can be based on a wider variety of approaches including integrated nested Laplace approximation methods or generalized additive models. Even when Markov chain Monte Carlo (MCMC) methods are used (as in [6]), marginal models avoid the need to sample unknown $y_{t,s}$ as a latent quantity, which can cause slow mixing and thus potentially increase the computation time required to achieve MCMC convergence and good‐quality samples (all else being equal).

Lacking an explicit posterior predictive distribution for the total counts, marginal models predict them through $y_{t,s}=\sum_{d=0}^{D_{\max}}z_{t,d,s}$. For instance, if only $z_{t,0,s}$ is observed, it can be added to posterior predictive simulations of the not‐yet‐observed $z_{t,d,s}$ (d>0). The predictive variance of $y_{t,s}$ will naturally be more concentrated for t where more $z_{t,d,s}$ are observed. Stoner and Economou [7] argued that reconstructing an accurate and precise predictive distribution for $y_{t,s}$ through the summation of predicted $z_{t,d,s}$ relies on capturing the covariance structure of $z_{t,s}$ accurately. Since published marginal models assume that $z_{t,d,s}$ are independent and identically distributed conditional on latent and covariate effects, they implicitly rely on those effects and their functional relationship to the mean, to capture covariance across delays. Notably, the NobBS approach has no delay effects that vary over time and thus assumes that the average/expected characteristics of the delay distribution are homogeneous over the time period to which the model is fit. Instead, the approach relies on fitting the model to a “moving window” subset of the data–such that the parameters (e.g., $\beta_{s}$) can evolve with ongoing use of the model over time–to match the properties of recent data better. In practice, a shorter window allows more sensitivity to changes over time in the delay distribution, but could make parameter estimates more volatile because the training data set is smaller [6].

Despite the use of moving windows to allow for some change over time in the delay distribution, the NobBS model still implies that all within‐window systematic and random variability associated with reporting delays must be absorbed by the i.i.d. negative‐binomial variance. The Bastos et al. [4] model, in contrast, includes delay‐time interaction terms $\gamma_{t,d}$ that can capture systematic changes in the delay distribution over time. Since $\gamma_{t,d}$ are first‐order random walks for each delay, they are arguably flexible enough to capture less persistent/structured variability in the delay distribution as well. However, a key limitation of the approach proposed by [4] is that delay effects like $\gamma_{t,d}$ are not structured in a way that recognizes that the delay distribution is compositional in nature. In other words, their approach does not recognize that the share of $y_{t,s}$ reported at each delay sums to 100% over all delays $d\leq D_{max}$. For instance, where/when reporting is faster, effects for earlier delays should increase and effects for later ones should decrease. However, no such behavior is enforced or encouraged in the Bastos et al. [4] approach and so there is no guarantee that the predictive distribution for $y_{t,s}$ will be optimally precise or accurate. Notably, when nowcasting, and even more so when forecasting, uncertainty in delay effects that have no compositional constraint or covariance across delay can propagate into excessive predictive uncertainty for $y_{t,s}$ [7]. Bergström et al. [9] also noted that, in the Bastos et al. [4] approach, “there is no clear separation between a model for the time trend in total counts and the (time‐varying) reporting delay distribution.”

Finally, another family of marginal models that potentially addresses these specific concerns comprises those based on [10]. The most recent of these, [11] and [9], propose a negative‐binomial model for the counts reported at each delay $z_{t,d,s}$ where the mean is expressed as the product of the expected proportion of cases reported at each delay, $p_{t,d,s}$, and the expected disease incidence, $\lambda_{t,s}$:

(7) $$\begin{array}{l} z_{t,d,s}|p_{t,d,s},\lambda_{t,s},\theta_{s}∼\text{Negative‐Binomial}(p_{t,d,s}\lambda_{t,s},\theta_{s}), \end{array}$$

where $\log(\lambda_{t,s})$ is a first‐order random walk plus the possible addition of covariate effects. The expected proportions reported at each delay $p_{t,d,s}$ are modeled via a hazard‐like structure:

(8) $$\begin{array}{ll} \log\left(\frac{h_{t,d,s}}{1-h_{t,d,s}}\right) & =\gamma_{d,s}+W_{t,d,s}^{\prime }\eta_{s};\; \end{array}$$

(9) $$\begin{array}{ll} p_{t,0,s} & =h_{t,0,s},\; \end{array}$$

(10) $$\begin{array}{ll} & \ldots \; \end{array}$$

(11) $$\begin{array}{ll} p_{t,d,s} & =h_{t,d,s}\left(1-\overset{d-1}{\underset{j=0}{\sum }}p_{t,d,s}\right),\; \end{array}$$

where effects $W_{t,d,s}^{\prime }\eta_{s}$ determine changes over time in $p_{t,d,s}$. That is, the model estimates effects on the fraction of not‐yet‐reported cases that we expect to be reported at delay d ($h_{t,d,s}$).

This approach therefore allows the average/expected characteristics of the delay distribution to change over time via $W_{t,d,s}^{\prime }\eta_{s}$–which in [11] and [9] are linear effects of time with break points every two weeks–and so it is more temporally flexible than [6]. Also, unlike in [4], it separates delay effects from total disease incidence effects and treats them as proportions that sum to 100% over delays.

Nevertheless, all these marginal approaches share a common limitation in our view: They rely on the i.i.d. negative‐binomial to capture random variability in the delay distribution that can't be explained by structured effects (e.g., linear effects with break points). Since the i.i.d. negative‐binomial variance of the model is not correlated across delays, the predictive distribution for nowcasted/forecasted $y_{t,s}$ based on summing predicted $z_{t,d,s}$ may not have the correct variance or uncertainty.

In essence, since marginal models do not explicitly model the total counts $y_{t,s}$, great care must be taken to ensure that the mean structure and latent effects can implicitly recover a suitable predictive distribution for $y_{t,s}$ through the summation of predicted $z_{t,d,s}$. In the next subsection, we aim to explain how a conditional/hierarchical approach can offer a more rigorous treatment of the various sources of variability in the data.

### 2.2 The GDM Method

Viewing delayed reporting as a challenge of capturing all the main sources of variability in the total counts and delay distribution motivates an approach based on the GDM family of distributions. Stoner and Economou [7] proposed a conditional GDM model for the partial counts $z_{t,s}$ given the totals $y_{t,s}$, which are modeled using a negative‐binomial distribution:

(12) $$\begin{array}{ll} y_{t,s}|\lambda_{t,s},\theta_{s} & ∼\text{Negative‐Binomial}(\lambda_{t,s},\theta_{s}), \end{array}$$

(13) $$\begin{array}{ll} z_{t,s}|\nu_{t,s},\phi_{s},y_{t,s} & ∼\text{GDM}(\nu_{t,s},\phi_{s},y_{t,s}). \end{array}$$

The GDM is a conditional multinomial ($p_{t,s},y_{t,s}$) distribution, where the conditional mean proportion of $y_{t,s}$ reported at each delay is modeled by $p_{t,s}|\nu_{t,s},\phi_{s}∼\text{generalized‐Dirichlet}(\nu_{t,s},\phi_{s})$. In the conditional Multinomial, the mean, variance, and covariance are all fixed given $p_{t,s}$ and $y_{t,s}$. Therefore, the role of the generalized‐Dirichlet (GD) is to introduce extra random delay variability, tuned by concentration parameters $\phi_{s}>0$ to fit different delay patterns.

Stoner and Economou [7] argue that this additional flexibility improves predictive performance (of $y_{t,s}|z_{t,s}$) both theoretically and in demonstrated application. From a general statistical perspective, one can place the GDM on a complexity spectrum of multinomial mixture models. In the simplest end of the spectrum, the multinomial has no free variance parameters. Then, the Dirichlet‐multinomial has one free variance parameter, while the GDM has $\dim(z_{t,s})-1$ free parameters to control the variance ($\phi_{d,s}$). A further step up in complexity is the logistic‐normal‐multinomial (LNM), proposed in [12], which assumes a multivariate‐normal distribution for the Multinomial probabilities $p_{t,s}$ at an additive log‐ratio (ALR) transformed scale, that is, $\text{ALR}(p_{t,s})|\mu_{t,s},\sum_{s}∼\text{Multivariate‐Normal}(\mu_{t,s},\sum_{s})$. The LNM is arguably more flexible than the GDM, since it has a full covariance matrix $\sum_{s}$ to describe extra variability in the multinomial. However, learning or inferring $\sum_{s}$ can be a practical challenge since the number of degrees of freedom scales quadratically with $\dim(z_{t,s})$ [13]. Meanwhile, the number of parameters in the GDM scales linearly with $\dim(z_{t,s})$ and so it arguably sits in a flexibility “sweet spot” for general compositional count data regression problems, both in cases where the total $y_{t,s}$ is fixed/known, or where the total is an unknown quantity to be predicted, as is the case for correcting delayed reporting.

A useful representation of the GDM is as a product of conditional beta‐binomial distributions for the partial counts $z_{t,d,s}$ given the already observed partial counts $z_{t,0,s},\ldots ,z_{t,d-1,s}$ and the total counts $y_{t,s}$:

(14) $$z_{t,d,s}|\nu_{t,d,s},\phi_{d,s},N_{t,d,s}∼\text{Beta‐Binomial}(\nu_{t,d,s},\phi_{d,s},N_{t,d,s}).$$

Here, $N_{t,d,s}=y_{t,s}-\sum_{j=0}^{d-1}z_{t,j,s}$ is the remaining portion of $y_{t,s}$ yet to be reported as of delay d. This formulation is used in practice as a straightforward implementation of the GDM using MCMC where there are missing values in $z_{t,s}$ (as is the case with delayed reporting). The means of the beta‐binomials (and implicitly the means of the GDM) are controlled by parameters $\nu_{t,d,s}\in (0,1)$, which we call the “relative proportions” and are the expected fraction of the not‐yet‐reported total $N_{t,d,s}$ reported at delay d, that is, $\nu_{t,d,s}=E\left[z_{t,d,s}/N_{t,d,s}\right]$. The relative proportions $\nu_{t,d,s}$ in the GDM method are analogous to the quantities $h_{t,d,s}$ in ((8), (9), (10), (11)) from [11] and [9].

Meanwhile, concentration parameters $\phi_{d,s}>0$ control the extra variance of the beta‐binomial components–relative to binomial variance in the limit $\phi_{d,s}\to \infty$–allowing for a flexible covariance structure in $z_{t,s}$. The concentration parameters can also, in principle, be modeled as a general function of time, space, and delay $\log(\phi_{t,d,s})=g(t,d,s)$–although this has not yet been fully explored.

The GDM approach was first applied to dengue fever cases in [7]. Since then, it has been adopted by others, including for nowcasting COVID‐19 deaths in England [14] and for nowcasting COVID‐19 cases in Ohio [15]. Meanwhile, [1] presented an extensive comparison of the predictive performance of the GDM and the models proposed in [4] and [6] through a 15‐month rolling experiment predicting COVID‐19 hospital deaths in England. By separating and modeling all the key sources of variability, the GDM model was able to achieve the most accurate and the most precise predictions, on average.

Given its stronger theoretical and real‐world performance, and given the modularity of the Bayesian hierarchical approach, we feel the GDM is the most compelling choice of foundation for building an extended framework for joint nowcasting of related outcomes, diseases or strains–in this case, to improve nowcasting of SARI and COVID‐positive SARI in Brazil through realistic constraints and pooling information.

## 3 Methodology

### 3.1 Data

We downloaded individual SARI patient‐level data for 2021–2024 (26th June 2025 version) from the “Severe Acute Respiratory Syndrome Database–including COVID‐19 data” page (authors' translation) on the Brazilian Ministry of Health's OpenDataSUS website [16]. Among the dataset's 194 columns, some describe each patient's characteristics (e.g., age, sex, municipality of residence, vaccination status, risk factors), others relate to diagnostic testing and hospitalization details, and there are numerous date fields that together trace the timeline of the case from symptom onset through hospitalization to case closure.

For the purpose of disease surveillance, we take the date of first symptoms (DT_SIN_PRI) to be the case occurrence/onset date. We then take the date the record was entered into the digital system (DT_DIGITA) to be the SARI reporting date, as this is an automated date for when the case first becomes visible at a national level. The median delay from SARI onset to reporting is 11 days.

The columns AN_SARS2 and PCR_SARS2 indicate positive antigen and polymerase chain reaction (PCR) test results for the SARS‐CoV‐2 virus, respectively, and the corresponding columns DT_RES_AN and DT_PCR detail the day on which the test results were obtained. Here, we count a patient as COVID‐positive if they obtained either a positive antigen or positive PCR result for SARS‐CoV‐2. We take the first COVID‐positive date from DT_RES_AN or DT_RES_PCR, depending on which test yielded a positive result, or the earliest of the two where both tests returned a positive result.

For 80% of patients, the first positive test result date is earlier than the SARI reporting date (DT_DIGITA) and, for a further 6% of patients, the two dates are the same. For all other patients, the first positive date is after the SARI reporting date. However, there is no column that directly captures when the COVID‐positive test result first appeared in the electronic record or national database. For current operational surveillance, the date can be learned by comparing daily/weekly snapshots of the data. For our retrospective study, we do not have access to historic snapshots and so we opt instead to make assumptions that lead to a “minimal COVID‐positive delay” scenario and an “additional COVID‐positive delay” scenario. For the minimal delay scenario, we assume that the COVID‐positive reporting date is either the first positive test result date or the SARI reporting date (DT_DIGITA), whichever is latest–we believe this is the earliest that the COVID‐positive test result could theoretically appear in the national data.

For the additional delay scenario, we also consider the case closure date (DT_ENCERRA), defined as the date when the final SARI case diagnosis (CLASI_FIN) is completed (e.g., due to COVID‐19, influenza, another respiratory virus, another etiological agent, or not specified). The median delay between the SARI reporting date (DT_DIGITA) and the case closure date is 6 days, suggesting that the latter may capture potential extra delays before COVID‐positive test results are reported. However, this date has limitations: it may itself be entered with additional delay, and it is missing for 3.2% of cases with a COVID‐positive antigen or PCR result. For these reasons, in the additional delay scenario we assume that the COVID‐positive reporting date is the latest of the case closure date (when available), the first positive test result date, or the SARI reporting date.

**FIGURE 2:**
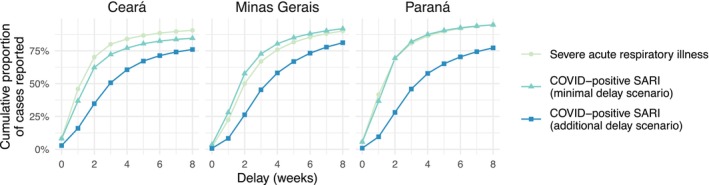
Cumulative proportion of severe acute respiratory illness (SARI) and COVID‐positive SARI cases reported by delay in weeks, in total across all available data 2021–2024, for the federative units of Ceará, Minas Gerais, and Paraná. Triangles show the cumulative reporting proportions for COVID‐positive SARI cases under the assumed minimal delay scenario and squares show the cumulative proportions under the additional delay scenario–both are explained in Section 3.1.

We used functions from the nowcaster package [17] for R to format patient‐level data into counts occurring each week by delay. Figure 2 shows the cumulative proportion of cases reported after each delay for SARI and COVID‐positive SARI under the two assumed scenarios, for three example federative units of Brazil. Note that, under the minimal COVID‐positive delay scenario, reporting of COVID‐positive SARI can be faster than the overall pattern for all SARI cases, for example, as shown in Figure 2 for Minas Gerais. Under the additional delay scenario, however, reporting of COVID‐positive SARI tends to lag behind reporting of total SARI considerably (lower cumulative proportions reported at each delay). For instance, a cumulative 86.4% of SARI cases were reported after a delay of 4 weeks in Paraná, compared to only 57.8% of COVID‐positive cases under the additional delay scenario.

Later, we apply models to both scenarios under the premise that if our proposed model consistently improves prediction accuracy, it is more likely to achieve real‐world impact in Brazil when applied to actual COVID‐positive reporting dates obtained using a data snapshot comparison strategy. We also present prediction performance results for all SARI ($y_{t,s}$) that do not rely on this premise.

To obtain an observed historical series of total SARI/COVID‐positive cases, it is essential, as in other nowcasting works (e.g., [6]), to assume a maximum delay. Here, we assume a maximum delay of 20 weeks (d=0,…,20), because 96.9% of all SARI cases, 97.4% of all COVID‐positive cases under the minimal delay scenario, and 92.8% of COVID‐positive cases under the additional delay scenario were reported within 20 weeks. The implication of this assumption is that predictions from the model will be slightly lower than the eventual total, for example, after a year or more.

Note that our definition of a COVID‐positive SARI patient–any patient with either a positive antigen or PCR test result recorded–differs from the final case diagnosis column (CLASI_FIN) and will thus yield different case counts. In total across the four years, our definition yields about 1.2 million COVID‐positive cases, compared to about 1.5 million with a final COVID‐19 case classification. We choose to use the two test result columns directly over the final classification on the basis that we assume the test results are available earlier than the final classification and would thus provide more timely information in a nowcasting model. This strategy could be adjusted in real‐world use, if needed.

### 3.2 Joint Model

For our proposed joint model, we begin by assuming a negative‐binomial model for the total SARI cases $y_{t,s}$ and a conditional GDM model for the partial counts $z_{d,s}$ expressed in terms of the conditional beta‐binomial series:

(15) $$\begin{array}{ll} y_{t,s}|\lambda_{t,s},\theta_{s} & ∼\text{Negative‐Binomial}(\lambda_{t,s},\theta_{s}), \end{array}$$

(16) $$\begin{array}{ll} z_{t,d,s}|\nu_{t,d,s}^{\left(z\right)},y_{t,s},\phi_{d,s}^{\left(z\right)} & ∼\text{Beta‐Binomial}(\nu_{t,d,s}^{\left(z\right)},\phi_{d,s}^{\left(z\right)},y_{t,s}). \end{array}$$

This is the GDM method for correcting reported delays, as described in Section 2.2 and originally presented in [7]. Within the general GDM approach, we need to choose how to capture systematic change over time (temporal structure) in $y_{t,s}$, that is, change in the incidence of SARI in each federative unit. Previously published nowcasting models have mainly employed first‐order random walks (e.g., [4, 6]) or splines (e.g., [1, 10, 14]), together with seasonal effects where appropriate (e.g., [4, 18]). Kline et al. [15] proposed using a semi‐local linear trend random effect, which we adopt here for its generality:

(17) $$\begin{array}{ll} \log(\lambda_{t,s}) & =\alpha_{t,s}, \end{array}$$

(18) $$\begin{array}{ll} \alpha_{t,s}|\alpha_{t-1,s},\zeta_{t-1,s},\sigma_{s}^{\left(\alpha \right)} & ∼\text{Normal}\left(\alpha_{t-1,s}+\zeta_{t-1,s},{\left(\sigma_{s}^{\left(\alpha \right)}\right)}^{2}\right), \end{array}$$

(19) $$\begin{array}{ll} \zeta_{t,s}|\rho_{s}^{\left(\zeta \right)},\zeta_{t-1,s},\sigma_{s}^{\left(\zeta \right)}, & ∼\text{Normal}\left(\rho_{s}^{\left(\zeta \right)}\zeta_{t-1,s},{\left(\sigma_{s}^{\left(\zeta \right)}\right)}^{2}\right). \end{array}$$

As $\sigma_{s}^{\left(\alpha \right)}\to 0$, $\alpha_{t,s}$ approaches a second‐order random walk with an autoregressive slope, and as $\sigma_{s}^{\left(\zeta \right)}\to 0$, $\alpha_{t,s}$ reduces to a first‐order random walk. As such, rather than assuming either a highly responsive model with little memory of an underlying trend (e.g., a first‐order random walk), or a smoother trend model that risks missing short‐term fluctuations (e.g., a second‐order random walk or a spline), the semi‐local linear trend model can balance the two dynamics, potentially capturing both short‐term (or sudden) changes and persistent trends that can be informative for nowcasting and forecasting. When forecasting future values of $\alpha_{t,s}$, any trend will initially persist and then level off at a rate determined by $\rho_{s}^{\left(\zeta \right)}$. This prevents upward slopes persisting as indefinite exponential growth in $\lambda_{t,s}=\exp(\alpha_{t,s})$ when forecasting.

To capture change over time in the delay distribution, we assume a hazard‐style structure, with a direct relationship between latent effect(s) and the fraction of the not‐yet‐reported total that we expect to be reported at delay d, which in this case is given by the beta‐binomial mean parameter $\nu_{t,d,s}^{\left(z\right)}$. This is the “hazard” version of the GDM approach, proposed in [7], and is analogous to the hazard structure ((8), (9), (10), (11)) assumed in [11] and [9]. Here, we assume that each $\nu_{t,d,s}^{\left(z\right)}$ can be explained by a simple first‐order random walk over time:

(20) $$\begin{array}{ll} \log\left(\frac{\nu_{t,d,s}^{\left(z\right)}}{1-\nu_{t,d,s}^{\left(z\right)}}\right) & =\eta_{t,d,s}^{\left(z\right)}, \end{array}$$

(21) $$\begin{array}{ll} \eta_{t,d,s}^{\left(z\right)}|\eta_{t-1,d,s}^{\left(z\right)},\tau_{d,s}^{\left(z\right)} & ∼\text{Normal}\left(\eta_{t-1,d,s}^{\left(z\right)},{\left(\tau_{d,s}^{\left(z\right)}\right)}^{2}\right). \end{array}$$

Note that the hazard version of the GDM can be seen as a direct alternative to methods similar to [11] and [9], where an i.i.d. NB model for $z_{t,d,s}$ is replaced by an NB model for $y_{t,s}$ and a conditional GDM for $z_{t,s}$, thus separating unstructured variability in disease incidence from unstructured variability in the delay distribution.

So far, we have specified a model that could be used to nowcast total SARI cases or another single disease/outcome. To extend this into a joint model for simultaneous nowcasting of all SARI and COVID‐positive SARI, we assume a beta‐binomial model for the number of SARI cases that test positive for COVID‐19, which we denote $x_{t,s}$, conditional on the total number of SARI cases $y_{t,s}$:

(22) $$x_{t,s}|\mu_{t,s},\chi_{s},y_{t,s}∼\text{Beta‐Binomial}(\mu_{t,s},\chi_{s},y_{t,s}).$$

The conditional beta‐binomial assumption ensures that the predicted $x_{t,s}$ are always less than or equal to corresponding $y_{t,s}$ and enables two‐way Bayesian information sharing between data on $x_{t,s}$ and $y_{t,s}$. We choose the beta‐binomial because it offers additional variance relative to the binomial, tuned by concentration parameter $\chi_{s}>0$, for example, to account for unmeasured covariate effects. Here we assume that the mean proportion of SARI cases that test positive for COVID‐19, $\mu_{t,s}$, evolves over time with another semi‐local linear trend random effect $\beta_{t,s}$–the same setup as in ((17), (18), (19)) but with a logit link function for $\mu_{t,s}$:

(23) $$\begin{array}{ll} \log\left(\frac{\mu_{t,s}}{1-\mu_{t,s}}\right) & =\beta_{t,s}, \end{array}$$

(24) $$\begin{array}{ll} \beta_{t,s}|\beta_{t-1,s},\omega_{t-1,s},\sigma_{s}^{\left(\beta \right)} & ∼\text{Normal}\left(\beta_{t-1,s}+\omega_{t-1,s},{\left(\sigma_{s}^{\left(\beta \right)}\right)}^{2}\right), \end{array}$$

(25) $$\begin{array}{ll} \omega_{t,s}|\rho_{s}^{\left(\omega \right)},\omega_{t-1,s},\sigma_{s}^{\left(\omega \right)} & ∼\text{Normal}\left(\rho_{s}^{\left(\omega \right)}\omega_{t-1,s},{\left(\sigma_{s}^{\left(\omega \right)}\right)}^{2}\right). \end{array}$$

As with the total SARI case counts, the COVID‐positive SARI case counts $x_{t,s}$ are not reported immediately and can be broken down into partial counts $c_{t,d,s}$, where $x_{t,s}=\sum_{d=0}^{D_{max}}c_{t,d,s}$. As such, we assume another conditional GDM model to capture the reporting delay distribution of the COVID‐positive SARI counts, with the same general setup as in (16), (20), and (21):

(26) $$\begin{array}{ll} c_{t,d,s}|\nu_{t,d,s}^{\left(c\right)},\phi_{d,s}^{\left(c\right)},x_{t,s} & ∼\text{Beta‐Binomial}\left(\nu_{t,d,s}^{\left(c\right)},\phi_{d,s}^{\left(c\right)},x_{t,s}\right), \end{array}$$

(27) $$\begin{array}{ll} \log\left(\frac{\nu_{t,d,s}^{\left(c\right)}}{1-\nu_{t,d,s}^{\left(c\right)}}\right) & =\eta_{t,d,s}^{\left(c\right)}, \end{array}$$

(28) $$\begin{array}{ll} \eta_{t,d,s}^{\left(c\right)}|\eta_{t-1,d,s}^{\left(c\right)},\tau_{d,s}^{\left(c\right)} & ∼\text{Normal}\left(\eta_{t-1,d,s}^{\left(c\right)},{\left(\tau_{d,s}^{\left(c\right)}\right)}^{2}\right). \end{array}$$

In summary, our proposed joint model comprises (i) an NB model for the total SARI cases $y_{t,s}$, (ii) a GDM model conditional on $y_{t,s}$ for the partial SARI counts broken down by delay $z_{t,d,s}$, (iii) a beta‐binomial model given $y_{t,s}$ for the total COVID‐positive SARI cases $x_{t,s}$, and (iv) a GDM model given $x_{t,s}$ for the partial COVID‐positive counts broken down by delay, $c_{t,d,s}$. This is not the first Bayesian nowcasting model for two diseases/outcomes–for instance, [19] proposed a model for dengue and chikungunya cases based on [4], where some effects are shared across both diseases–but it is likely the first where one set of cases is a subset of the other, and where this relationship is modeled directly, in a constrained hierarchical manner.

In the next section, we will investigate whether our proposed joint approach yields improvements in prediction accuracy compared to independent NB‐GDM models for SARI and COVID‐positive SARI and compared to independent models based on the popular “NobBS” method proposed by [6].

### 3.3 Prior Distributions and Implementation

Table 1 details the prior distributions that we assume for our proposed joint model. Our general approach is to specify priors that constrain the parameter space to “reasonable values” [20], informed by the SARI data while remaining sufficiently flexible to facilitate application to other diseases or regions. For instance, a value of $\sigma_{s}^{\left(\alpha \right)}=10$ would imply that a weekly multiplicative increase or decrease in disease incidence of exp(10)≈22000 is not extreme. We believe this is not “reasonable” for most disease data that we can imagine, and so we opt for a prior that implies it is very unlikely.

**TABLE 1:** Prior distributions for parameters in our proposed joint model and independent NB‐GDMs. Note that the gamma distribution is parameterised in terms of shape and rate.

Parameters	Prior distribution
$\alpha_{1,s},\beta_{1,s},\eta_{1,d,s}^{\left(z\right)},\eta_{1,d,s}^{\left(c\right)}$	$\text{Normal}(0,10^{2})$
$\zeta_{1,s},\omega_{1,s}$	$\text{Normal}(0,1^{2})$
$\sigma_{s}^{\left(\alpha \right)},\sigma_{s}^{\left(\beta \right)},\tau_{d,s}^{\left(z\right)},\tau_{d,s}^{\left(c\right)}$	Half‐normal$\left(0,1^{2}\right)$
$\sigma_{s}^{\left(\zeta \right)},\sigma_{s}^{\left(\omega \right)}$	Half‐normal$\left(0,0.1^{2}\right)$
$\rho_{s}^{\left(\zeta \right)},\rho_{s}^{\left(\omega \right)}$	Beta(4,1)
$\theta_{s},\chi_{s},\phi_{d,s}^{\left(z\right)},\phi_{d,s}^{\left(c\right)}$	Gamma(2,0.05)

Meanwhile, the Gamma(2,0.05) priors are centered around values for $\theta_{s}$, $\chi_{s}$, $\phi_{d,s}^{\left(z\right)}$, and $\phi_{d,s}^{\left(c\right)}$ that correspond to a moderate degree of overdispersion with respect to the Poisson and binomial distributions (depending on the values of $\lambda_{t,s}$, $\mu_{t,s}$, $\nu_{t,d,s}^{\left(z\right)}$, and $\nu_{t,d,s}^{\left(c\right)}$), while still allowing for much higher overdispersion (e.g., $\theta_{s}=5$) or more Poisson‐ or binomial‐like behavior (e.g., $\theta_{s}=100$).

Arguably, the most informative priors that we assume are Beta(4,1) priors for the autoregressive coefficients $\rho_{s}^{\left(\zeta \right)}$ and $\rho_{s}^{\left(\omega \right)}$. As ρ→1, the corresponding prior model for $\alpha_{t,s}$ or $\beta_{t,s}$ reduces to a local linear trend effect with more persistent trends. Conversely, as ρ→0, the prior model reduces to a first order random walk, with no memory of local trends. Since most diseases show trend dynamics from, for example, outbreaks, seasonality, or interventions, we opt to choose a prior that has more density for higher values of ρ. The 2.5% quantile of Beta(4,1) is 0.40, reflecting our belief that small values of ρ–where much more than half of the trend is “forgotten” after one time step (week)–are very unlikely. Meanwhile, the 97.5% quantile is 0.99, such that we are not ruling out high values of ρ for situations where diseases have smoother, more predictable trajectories.

We did not choose or alter any priors to optimize predictive performance in this application or to yield more favorable results against competitors.

As discussed in [7], we do not need to explicitly model partial counts $z_{t,d,s}$ or $c_{t,d,s}$ for all delays $d=0,\ldots ,D_{max}$. Instead, we can choose to include conditional beta‐binomials (as in (16)) for only $d=0,\ldots ,D^{\prime }$, where $0\leq D^{\prime }<D_{max}$. Note that the full model is specified by $D^{\prime }=D_{max}-1$, because $z_{t,D_{max},s}$ is then defined deterministically given $z_{t,d,s}$ for previous delays as $z_{t,D_{max},s}=y_{t,s}-\sum_{d=0}^{D_{max}-1}z_{t,d,s}$. In an experiment based on their model for dengue cases, [7] found that, all else being equal, the MCMC computation time was proportional to $D^{\prime }$, for example, the model took about 20 min with $D^{\prime }=4$ and about 40 min with $D^{\prime }=8$ (noting that their data did not include d=0).

For their application, they found that the choice of $D^{\prime }$ only appeared to affect accuracy and precision for predictions more than $D^{\prime }$ weeks before the nowcast date. They linked this finding, in general terms, to the fact that this is the period for which delayed counts $z_{t,d,s}$ for $d>D^{\prime }$ would actually be observed and would thus provide additional information to a model that included them. They concluded that the choice of $D^{\prime }$ can be viewed as a trade‐off between computation time and how far into the past predictions are required to be as precise as possible.

One way to choose $D^{\prime }$ is to select a value such that total counts up to $D^{\prime }$ weeks ago are already mostly reported, and so predictive uncertainty is already small. Here, we choose $D^{\prime }=5$ (i.e., we include beta‐binomials for d=0,…,5) noting that, historically, 88.4% of all SARI cases that will be reported within 20 weeks are reported within 5 weeks. Assuming a similar pattern to the experiment in [7] would suggest that choosing $D^{\prime }=5$ reduces the run time by about two‐thirds compared to the fully specified model ($D^{\prime }=D_{max}-1$) at the cost of increased uncertainty in the remaining ≈10% of cases reported after a delay of more than 5 weeks.

All models are fit to 52‐week moving windows; rather than fitting models to the whole time series of historical data, that is, we disregarded historical data before the 52‐week window. Moving windows are common in recent literature on disease nowcasting, for example, to improve computational feasibility in complex methods [1], or to keep estimation of time‐invariant model parameters relevant to more recent data [6].

We implement our proposed joint model in the statistical programming language R [21] and using the NIMBLE software package [22], which provides flexible implementations of MCMC algorithms for Bayesian inference. We use NIMBLE to run two MCMC chains per instance of our model for 70k iterations, discarding the first 20k iterations as burn‐in and applying a thinning interval of 20 to constrain system memory usage.

The general strategy that we employ for setting initial values is to randomly generate parameter values well within the bulk of their corresponding prior distributions, to avoid extreme combinations of initial values with very low posterior probabilities. Then, to assess convergence of the MCMC chains, we calculate the potential scale reduction factor (PSRF) for all posterior samples of $\lambda_{t,s}$, $\mu_{t,s}$, unknown $y_{t,s}$, and unknown $x_{t,s}$. By convention, if started from different initial values, we assume the chains have converged if the PSRF is less than 1.05 [23]. Across all joint models and independent NB‐GDMs (introduced in Section 3.4), 95.2% of the PSRF values are under 1.05, and 99.6% are under 1.20.

### 3.4 Rolling Prediction Experiment

To investigate the potential effectiveness of our proposed joint model for nowcasting and forecasting SARI and COVID‐positive SARI, we carried out a rolling prediction experiment. Here, we fit models to 20 nowcast dates at approximately equal intervals, from the 23rd of December 2021 up to the 1st of August 2024. For a realistic assessment, we need to fit models and generate predictions using only data that would have been available at each nowcasting date, $T_{\text{now}}$, which includes historical observed $y_{t,s}$ where $t+D_{\max}\leq T_{\text{now}}$ and observed $z_{t,d,s}$ where $t+d\leq T_{\text{now}}$. We fixed the nowcast dates prior to carrying out the experiment and did not investigate whether different dates would yield more favorable results.

Recall from Section 3.1 that we consider two alternative COVID‐positive reporting delay scenarios; a “minimal delay scenario” that we believe reflects the minimum possible COVID delay structure and an “additional delay scenario,” where we assume there is some additional delay after the COVID‐test results become available. This means that we have one set of $c_{t,d,s}$ and $x_{t,s}$ for each scenario ($x_{t,s}$ differ because the scenario slightly alters how many cases are reported before the maximum delay of 20 weeks) and we fit models separately to both data sets/scenarios.

We fit all models to data from each of the 27 federative units in Brazil, such that we have 20×27=540 sets of predictions for both SARI and COVID‐positive SARI. This allows us to assess their performance when applied to a wide variety of time series, each with different shapes and scales in the levels of SARI and COVID‐positive SARI incidence, and each with differing systematic delay mechanisms.

We predict cases up to a four week forecasting horizon ($T_{\text{now}}+4$). For each model fit, we obtained posterior predictive samples of recent $y_{t,s}$ and $x_{t,s}$ directly from the MCMC algorithm. We simulated posterior predictions of future $y_{t,s}$ and $x_{t,s}$ using the NB and beta‐binomial random generator functions, respectively, and using posterior samples of corresponding $\lambda_{t,s}$, $\mu_{t,s}$, $\theta_{s}$, and $\chi_{s}$.

For comparison, we fit models based on the “NobBS” approach proposed by [6], implemented in the NobBS R package [8], to the same 20 52‐week windows, under both COVID‐positive delay scenarios. We fit independent models to each region and to both count types (SARI and COVID‐positive SARI). We used all default model specification settings, except changing the likelihoods for $z_{t,d,s}$ and $c_{t,d,s}$ from Poisson to negative‐binomial and modifying the code to store samples of the NB inverse dispersion parameter. The NobBS package uses **rjags** [24] to generate posterior samples using MCMC; by default, one chain is run for 10k iterations, and 1k samples are discarded as burn‐in. To allow convergence checking, we run two chains for each instance of the model and, to achieve similar PSRF results to the GDM models, we increase the number of iterations to 20k, discarding the first 5k iterations as burn‐in and applying a thinning interval of 6 to conserve system memory. We compute the PSRF for all $\alpha_{t,s}$ and $\beta_{d,s}$, which together fully define the mean $\lambda_{t,d,s}$. Across all NobBS models, 95.5% of the PSRF values are under 1.05, and 97.8% are under 1.20.

Though the NobBS package does not have built‐in forecasting capabilities, we used Monte Carlo simulation to carry forward the first‐order random walks into the future and to simulate total SARI and COVID‐positive SARI forecasts from their respective posterior predictive distributions. Specifically, for each saved posterior/MCMC sample of the random walk for the nowcasting date, $\alpha_{T_{now},s}$, and corresponding sample of the random walk standard deviation $\sigma_{s}^{\left(\alpha \right)}$, we simulate once from $\text{Normal}\left(\alpha_{T_{now},s},{\left(\sigma_{s}^{\left(\alpha \right)}\right)}^{2}\right)$. This generates a posterior predictive sample of $\alpha_{T_{now}+1,s}$, which we can then use to simulate $\alpha_{T_{now}+2,s}$, and so on, up to the desired forecasting horizon. We can combine the simulated future $\alpha_{t,s}$ with the corresponding posterior sample of $\beta_{d,s}$ and $\theta_{s}$, to simulate each unobserved $z_{t,d,s}$ from $\text{Negative‐Binomial}(\lambda_{t,d,s},\theta_{s})$. Finally, we compute the predicted future case counts (i.e., total SARI or COVID‐positive SARI) as $y_{t,s}=\sum_{d=0}^{D_{max}}z_{t,d,s}$.

To further assess the utility of the joint modeling approach, we fit independent models for SARI and COVID‐positive SARI where we replace the beta‐binomial model for $x_{t,s}$ given $y_{t,s}$ in (22) with a negative‐binomial model that does not depend on $y_{t,s}$:

(29) $$x_{t,s}|\mu_{t,s},\chi_{s}∼\text{Negative ‐ Binomial}(\mu_{t,s},\chi_{s}).$$

Here, $\mu_{t,s}$ is now the mean number of COVID‐positive SARI cases–which we model at the log‐scale with a semi‐local linear trend model, as in (18) and (19)–and $\chi_{s}>0$ is an inverse dispersion parameter. In essence, though SARI and COVID‐positive SARI are fit together in the same hierarchical model for convenience, they share no parameters or dependence, and so they are predicted entirely independently. Henceforth, we will call these the “independent NB‐GDMs.” All priors and implementation for this approach are the same as in 3.3.

## 4 Results

We arrange predicted case counts by “prediction horizon”: the difference between the date that we are predicting the number of cases for and the nowcast date $T_{now}$ (the hypothetical present week we have data reported up to). As such, horizon <0 corresponds to predictions for weeks before $T_{now}$ where the cases are not yet fully reported; horizon =0 means contemporaneous nowcasts; and horizon >0 means forecasting.

To compare models and to assess how performance changes with respect to prediction horizon, we compute three summaries that capture accuracy/calibration/sharpness relative to the eventually reported totals:

- Mean absolute error (MAE) of the posterior median predictions.
- Mean continuous ranked probability score (CRPS) of all the posterior predictive samples.
- Root mean square error (RMSE) of the posterior median predictions.

MAE and RMSE both measure point prediction accuracy, while mean CRPS measures the quality of the entire posterior predictive distribution, capturing both calibration (statistical consistency with observations) and sharpness (concentration of the distribution)–where the goal is correct calibration and maximum sharpness without loss of calibration [25]. In short, MAE and RMSE evaluate accuracy of the models' “best” estimates, whereas CRPS is a probabilistic analogue that evaluates the full posterior predictive distributions.

We also report indicative computation times for each method. We run all models on an Apple M1 Max CPU (10 cores, 64 GB system memory), executing 20 nowcasts in parallel across 10 processing clusters. Within each cluster, we run the MCMC for the 27 federative units sequentially. For a straightforward comparison across methods, we calculate the mean time per nowcast as the total elapsed time (including NIMBLE model and MCMC compilation time) for all 20 nowcasts and 27 federative units, divided by the number of nowcasts.

**FIGURE 3:**
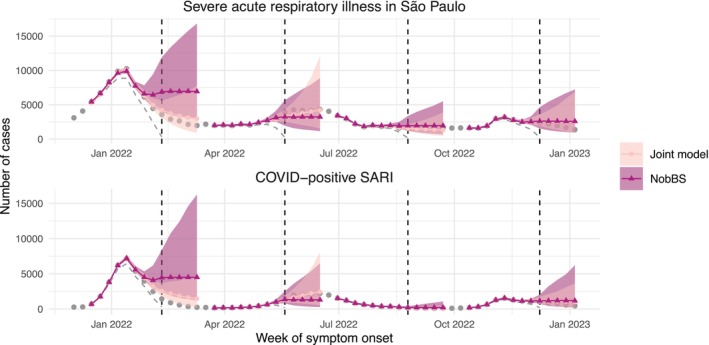
Comparison of nowcast and forecast predictions from the joint model and NobBS model, in the assumed minimal COVID‐19 test reporting delay scenario, for São Paulo on four example nowcast dates: 10th February 2022, 19th May 2022, 25th August 2022, and 8th December 2022 (vertical dashed lines). Grey points show the total number of cases with first symptom onset each week; grey dashed lines show the number of cases reported so far at the nowcast date, solid lines. Colored lines/points show posterior median predictions and shaded areas show associated 95% prediction intervals.

First, Figure 3 shows predictions for São Paulo for four example nowcast dates in 2022 (10th February, 19th May, 25th August, 8th December), from the joint model (Section 3.2) and from NobBS, in the minimal COVID‐positive reporting delay scenario. In a real‐world setting, we would not see the total number of cases occurring each week (the grey points). Meanwhile, the plotted model predictions (solid lines, shapes and shaded intervals) are indicative of the additional information offered over and above the total reported so far (decreasing grey dashed lines).

The main visible difference between the two approaches is that the joint model appears to capture and continue trends in the level of disease much more successfully, owing to the semi‐local linear trend effects (Section 3.2). With the NobBS approach, predictions level off in line with the first‐order random walk assumption. In these specific examples, we can see that, in general, the memory and continuation of trends in the joint model lead to more accurate predictions, not just for forecasting–for which NobBS was not strictly designed–but also for contemporaneous nowcasting.

**FIGURE 4:**
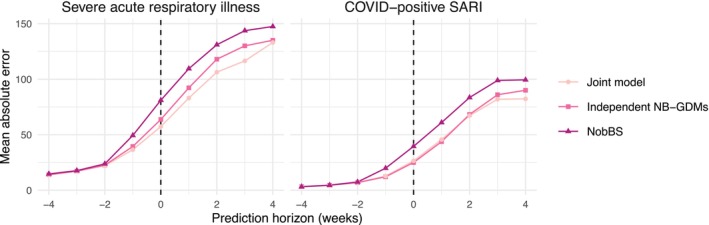
Mean absolute errors for the prediction of severe acute respiratory illness cases and the number of those testing positive for COVID‐19, across the 20 nowcast dates and across all 27 federative units of Brazil, in the assumed minimal COVID‐19 test reporting delay scenario. Prediction horizon is defined in Section 4.

Table 2 presents the three prediction performance summaries (MAE, RMSE, and mean CRPS) for contemporaneous nowcasting (horizon=0), across all 27 federative units, and provides broader evidence of improvement in prediction accuracy/calibration/sharpness compared to the mainstream competitor, NobBS. First, note that the mean compute time per nowcast is similar across the different methods and scenarios, about 7–9 min. However, when compared to NobBS the joint model has about a 27%–29% lower MAE (depending on the COVID‐positive delay scenario), a 28%–29% lower mean CRPS, and a 42%–44% lower RMSE for SARI cases. The joint model also has a 33%–39% lower MAE, a 34%–40% lower CRPS, and a 46%–47% lower RMSE for COVID‐positive SARI cases. We can also see a consistent improvement over NobBS for the minimal COVID‐positive delay scenario in Figure 4. Here, MAE increases as the prediction horizon increases, that is, forecasts are less accurate than contemporaneous nowcasts, though the improvement over NobBS is fairly stable from horizon=0 up to the forecast horizon of 4 weeks. The general pattern for the additional delay scenario (not presented) is the same, though showing a greater improvement over NobBS for COVID‐positive SARI.

**TABLE 2:** Comparison of model nowcasting performance metrics (mean absolute error, mean continuous ranked probability score (CRPS), root mean square error, mean compute time per nowcast) under the minimal and additional COVID‐positive test reporting delay scenarios, across all 27 federative units of Brazil. Green shaded cells indicate the best‐performing model in each case.

Scenario	Model	Mean absolute error		Mean CRPS		Root mean square error		Compute time (minutes)
		SARI	COVID‐ positive	SARI	COVID‐ positive	SARI	COVID‐ positive	
Minimal delay	Joint model	57.3	26.5	42.8	20.3	130.2	99.7	7.5
	Independent NB‐GDMs	63.8	25.0	47.3	19.1	159.1	86.7	7.3
	NobBS	81.1	39.5	60.3	30.6	233.6	186.0	8.6
Additional delay	Joint model	58.7	32.1	43.6	25.0	134.9	137.0	7.2
	Independent NB‐GDMs	64.8	31.3	48.1	23.9	165.3	113.1	7.2
	NobBS	80.9	52.9	60.3	41.5	232.9	256.5	8.7

The joint model is the most accurate, sharp, and best calibrated for predictions of total SARI cases at all prediction horizons (−4 weeks–4 weeks) and for forecasting COVID‐positive SARI 2+ weeks in the future, but the independent NB‐GDMs are marginally better for contemporaneous nowcasts of COVID‐positive SARI (Table 2) and forecasts one week ahead (Figure 4).

**FIGURE 5:**
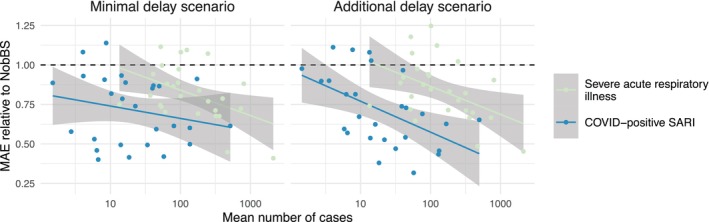
Mean absolute errors (MAE) of predictions from the joint model divided by MAEs of predictions from the “NobBS” model [6], by federative unit of Brazil, in both the assumed minimal COVID‐19 test reporting delay scenario and the additional delay scenario. MAEs are for contemporaneous nowcasting, that is, predicting for the current week using only data reported in that week or earlier. Lines show linear regression fits with 95% confidence intervals.

Our summaries in Table 2 and in Figure 4 are naturally dominated by high case counts, for example, from outbreaks or more populous federative units. Other options, such as mean absolute percentage error, may not have this characteristic. However, in the context of disease surveillance–where a human casualty is not worth less just because it occurs for example, in a more populous region–we agree with the argument posed by [26] that we ought to penalize prediction errors for higher cases/deaths more than for lower cases/deaths, where the relative error is the same. On that note, Figure 5 shows MAEs of predictions from the joint model divided by MAEs from NobBS, for contemporaneous nowcasting in each federative unit, arranged over the *x*‐axis by the mean number of cases in 2021–2024, in each federative unit. There is a clear pattern, especially for SARI and for COVID‐positive SARI in the additional delay scenario, that the relative improvement over NobBS is higher, on average, when the absolute level of disease in a region is higher. This implies that the case for our proposed joint model is strongest in regions with larger populations or higher overall infection risks.

Finally, while our chosen three summaries (MAE, RMSE, mean CRPS) evaluate marginal prediction accuracy/calibration/sharpness for either SARI or COVID‐positive SARI, we should also investigate the calibration and sharpness for joint prediction of both quantities together, for example, for planning against likely joint outcomes for SARI and COVID‐positive SARI incidence. To check this, we compute the energy score, a proper scoring rule that balances calibration and sharpness in the multivariate setting [25]. It is influenced by both marginal performance and the dependence structure between outcomes.

**FIGURE 6:**
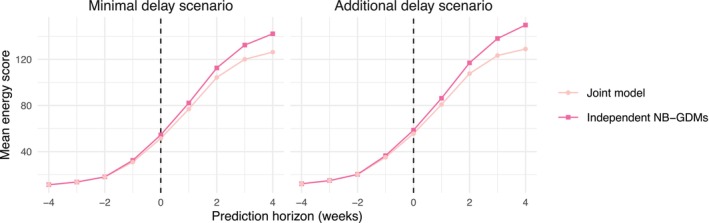
Mean energy score for the joint prediction of severe acute respiratory illness cases and the number of those testing positive for COVID‐19, across the 20 nowcast dates and across all 27 federative units of Brazil, under each COVID‐19 test reporting delay scenario. Prediction horizon is defined in Section 4.

Figure 6 shows the mean energy score for both sets of models, across all nowcast dates and federative units and by prediction horizon. Under both scenarios, the joint model yields an advantage for forecasting (lower energy score) that grows with prediction horizon. This suggests that the hierarchical structure of the joint model may provide more reliable projections of future incidence, reflecting its ability to share information between SARI and COVID‐positive SARI and to project trends more coherently.

## 5 Discussion

The emergence of SARS‐CoV‐2 (COVID‐19) has introduced additional complexity in the incidence and management of SARI cases in Brazil. Supplementing existing SARI surveillance with nowcasting of the number of cases caused by SARS‐CoV‐2–identified through positive test results–is valuable both as an indicator of COVID‐19 prevalence in the wider population (since more severe cases are more likely to be tested) and as a trigger for interventions such as targeted testing, outbreak investigation, or preparation of hospital resources. Current early warning systems in Brazil, however, model total SARI and COVID‐positive SARI separately, without an explicit hierarchical structure that captures their natural dependence and ensures coherence.

In this article, we developed a flexible framework for joint nowcasting and forecasting of two outcomes with a strict nesting structure, where one quantity is a subset of the other. To achieve this, we proposed a new hierarchical approach with its roots in the GDM method for correcting delayed reporting of a single disease or outcome. The key innovations were (i) explicitly modeling the hierarchical relationship between SARI cases and the subset of cases testing positive for COVID‐19, and (ii) specifying separate conditional GDM models for the reporting delays of each outcome, allowing for the different delay mechanisms associated with SARI and COVID‐positive SARI. Unlike most existing nowcasting approaches that focus solely on reconstructing the present or recent past, our framework also generated short‐term forecasts, to provide decision‐makers with both situational awareness (nowcasting) and early warning (forecasting). This required not only correcting for reporting delays to establish an accurate baseline, but also projecting trends forward in a plausible manner, which we achieved using semi‐local linear trend effects.

We evaluated our proposed framework against the widely cited “NobBS” approach of McGough et al. [6], and against an equivalent model without dependence between SARI and COVID‐positive SARI (“independent NB‐GDMs”), in a rolling prediction experiment. For contemporaneous nowcasting, the joint model achieved a 9%–10% reduction in MAE for SARI but a slightly higher MAE (3%–6%) for COVID‐positive SARI, compared with the independent NB‐GDMs. These findings subverted our expectations prior to carrying out this work: we anticipated that joint modeling would benefit prediction of COVID‐positive cases most due to the extra steps involved in obtaining test results (e.g., sample collection and analysis). Yet, joint modeling appeared to mainly benefit prediction of total SARI, even in the longer COVID test reporting delay scenario. A possible explanation is that modeling SARI together with its COVID‐positive subset helped stabilize total SARI predictions. Another possible explanation is that COVID‐positive predictions may not have benefited because their counts were smaller (particularly in the later years) and thus carried a weaker signal.

Altogether, although the gains from joint modeling were modest when looking at SARI and COVID‐positive prediction errors individually, our results suggest that it was the best overall approach, and even modest gains are valuable in disease surveillance, where improved accuracy can translate directly into more timely and reliable situational awareness. The joint model also provides a correlated predictive distribution for total SARI and COVID‐positive SARI; this guarantees coherence between the two quantities and enables decision‐makers to assess not only the marginal trajectories of each, but also the probability of joint outcomes (e.g., concurrent surges). In support of this, the joint model achieved lower energy scores for joint forecasting than the independent NB‐GDMs.

Both the joint and independent NB‐GDM models substantially outperformed the widely cited NobBS approach, achieving roughly one‐third lower MAE and CRPS and about 40% lower RMSE for nowcasting. These findings add to a growing body of evidence that explicitly modeling and disentangling the main sources of variability in delayed reporting yields superior predictive performance in practice.

For new applications, it can be challenging to know whether these gains justify additional complexity and computation without comparing alternative methods in out‐of‐sample experiments based on historical data. In this application, we found that the relative error reduction over NobBS increased with the mean number of cases in each region, which suggests that the flexibility of a hierarchical GDM‐based approach may be particularly advantageous when incidence is high and thus the underlying signals are clearer. From a public health perspective, this is encouraging, as it indicates our method may be especially useful during major outbreaks or in high‐burden regions, with the largest gains over alternative approaches occurring precisely where the need for accurate and timely predictions is greatest.

Since computation times were comparable with existing methods, the main obstacle to immediate operational implementation of our joint approach by public health authorities in Brazil is the absence of an unambiguous record of when COVID‐positive test results become available in the national database and, therefore, what the actual reporting delays are. Ideally, reporting processes could be amended to include this information. In the meantime, researchers at the Oswaldo Cruz Foundation have already developed a successful workaround involving comparisons of database snapshots to determine when COVID‐positive results first appear in the system. As this was not possible for the historical data we analyzed, we considered two contrasting COVID test delay scenarios. We believe this was a reasonable solution to maximize the generalizability of our findings to the real‐world delay pattern, especially since our models consistently outperformed a highly cited alternative under both scenarios.

Finally, while our approach was motivated by joint surveillance of SARI and COVID‐positive SARI cases in Brazil, it could readily be adapted for other settings. The number of subsets of the total outcome could be increased beyond a single subset by including additional conditional beta‐binomial components as in (22), effectively creating a GDM structure for the breakdown of total cases. For SARI, this would allow joint monitoring of cases attributed to multiple viruses (e.g., SARS, influenza, or respiratory syncytial virus). More generally, the same framework could be used to partition total cases by demographic characteristics (e.g., age group, sex) or by health outcome (e.g., cases requiring ventilation or resulting in death).

To facilitate wider adoption, our proposed approach will be included in an R package for nowcasting and forecasting single or joint disease outcomes that is currently under development. The R code published alongside this article can be modified for other datasets, but the package will make our approach more accessible through a one‐line interface, similar to NobBS and nowcaster. By lowering barriers to implementation, we hope to extend the benefits of joint modeling of related outcomes and of the GDM method to more disease surveillance applications.

In summary, our proposed framework delivered compelling results for SARI in Brazil and offers a flexible foundation for other joint disease surveillance applications.

## Funding

This work was supported by the Engineering and Physical Sciences Research Council (Grant No. EP/X525716/1) and HORIZON EUROPE Innovative Europe (Grant No. 856612). LSB is funded by FAPERJ (Grant No. E‐26/204.098/2024) and CNPq (Grant No. 302603/2025‐5).

## Conflicts of Interest

The authors declare no conflicts of interest.

## Supporting information


Data S1.

## Data Availability

The data that support the findings of this study are openly available in Banco de dados da Síndrome Respiratória Aguda Grave (SRAG) at https://opendatasus.saude.gov.br/en/dataset/srag‐2021‐a‐2024. At the time of writing, the SARI patient‐level data used in this work (26th June 2025 version) are publicly available on the Brazilian Ministry of Health's OpenDataSUS website [16]. All R code required to reproduce this work can be found in the online version of the article at the publisher's website.
