# Supplementary material for: Joint Bayesian Nowcasting of Severe Acute Respiratory Illness and COVID‐19 Positives in Brazil
Source: Stat Med. 2026 Apr 17;45:e70529. doi: 10.1002/sim.70529 (PMC13090138; doi:10.1002/sim.70529)
Supplement: Supplementary file 1 — Data S1. [file SIM-45-0-s001.zip › Software for joint Bayesian nowcasting/Plots/cumu_explore_plot.pdf]

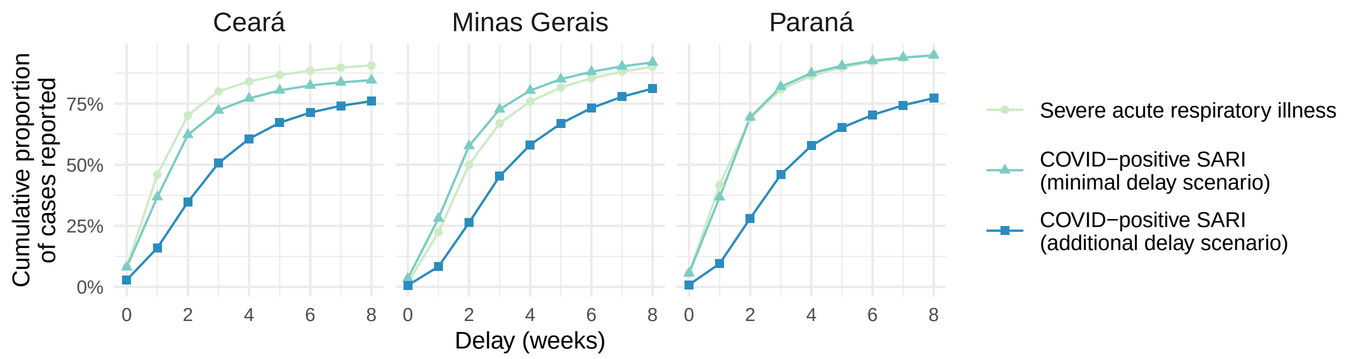
Ceará
Minas Gerais
Paraná
Cumulative proportion of cases reported
0%
25%
50%
75%
0
2
4
6
8
Delay (weeks)
Severe acute respiratory illness
COVID−positive SARI (minimal delay scenario)
COVID−positive SARI (additional delay scenario)
