# Supplementary material for: Joint Bayesian Nowcasting of Severe Acute Respiratory Illness and COVID‐19 Positives in Brazil
Source: Stat Med. 2026 Apr 17;45:e70529. doi: 10.1002/sim.70529 (PMC13090138; doi:10.1002/sim.70529)
Supplement: Supplementary file 1 — Data S1. [file SIM-45-0-s001.zip › Software for joint Bayesian nowcasting/Plots/example_preds_end.pdf]

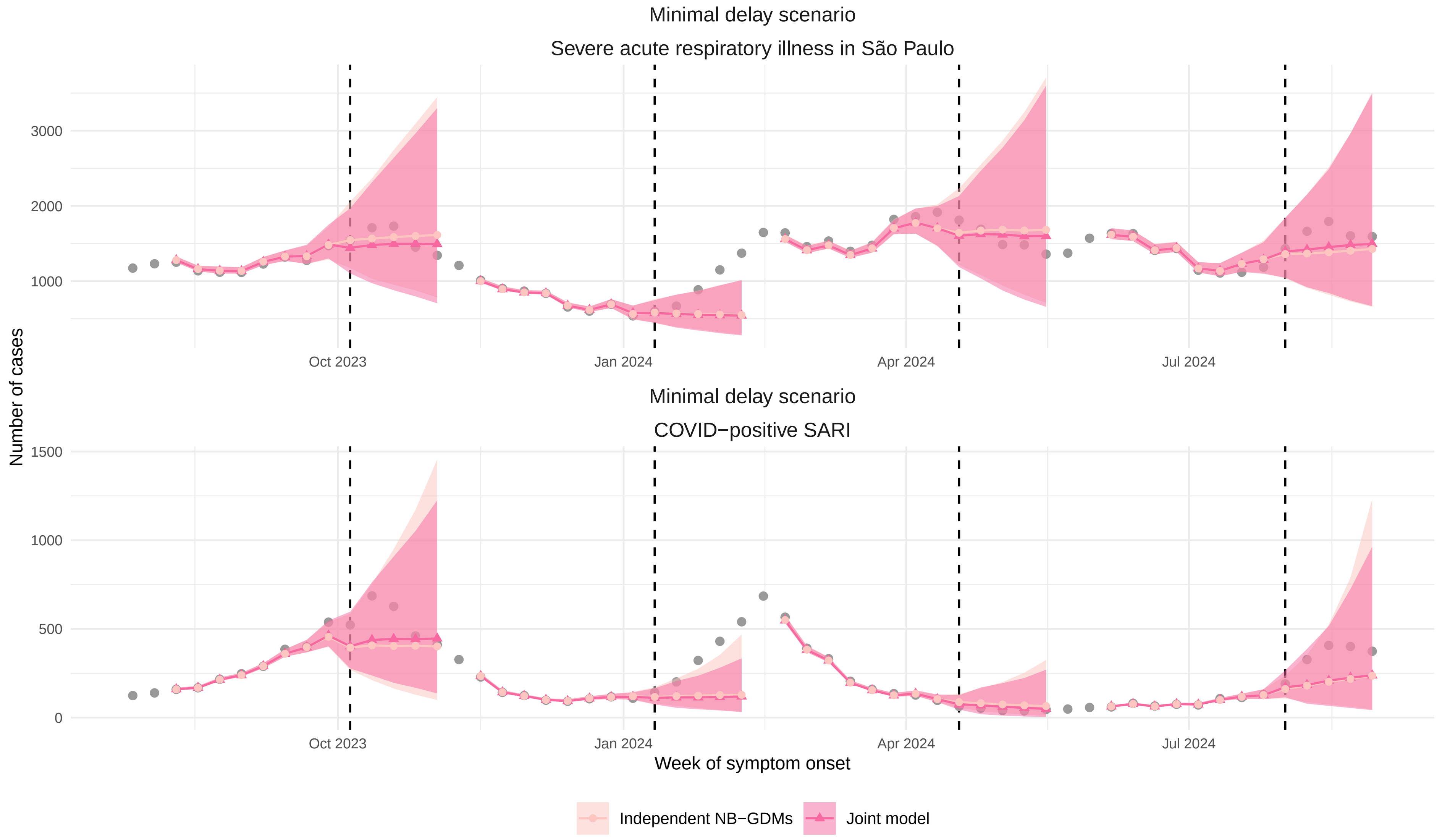

Minimal delay scenario
Severe acute respiratory illness in São Paulo
3000
2000
1000
Oct 2023
Jan 2024
Apr 2024
Jul 2024
Minimal delay scenario
COVID−positive SARI
1500
1000
500
0
Oct 2023
Jan 2024
Apr 2024
Jul 2024
Number of cases
Week of symptom onset
Independent NB−GDMs
Joint model
