# Supplementary material for: Joint Bayesian Nowcasting of Severe Acute Respiratory Illness and COVID‐19 Positives in Brazil
Source: Stat Med. 2026 Apr 17;45:e70529. doi: 10.1002/sim.70529 (PMC13090138; doi:10.1002/sim.70529)
Supplement: Supplementary file 1 — Data S1. [file SIM-45-0-s001.zip › Software for joint Bayesian nowcasting/Plots/example_preds_start.pdf]

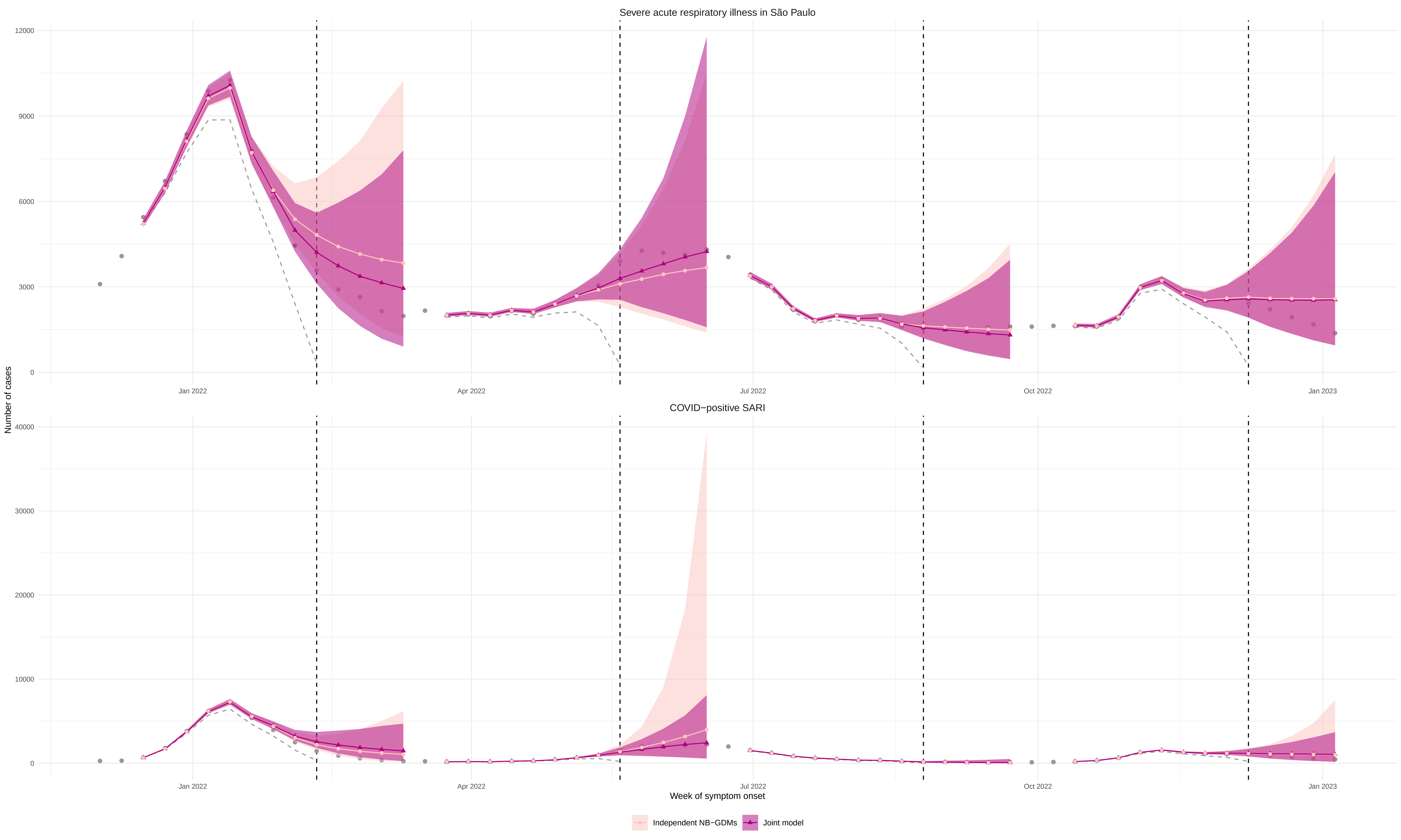

Severe acute respiratory illness in São Paulo
COVID−positive SARI
Number of cases
Week of symptom onset
0
3000
6000
9000
12000
10000
20000
30000
40000
Jan 2022
Apr 2022
Jul 2022
Oct 2022
Jan 2023
Independent NB−GDMs
Joint model
