# Supplementary figures and images for: Joint Bayesian Nowcasting of Severe Acute Respiratory Illness and COVID‐19 Positives in Brazil
Source: Stat Med. 2026 Apr 17;45:e70529. doi: 10.1002/sim.70529 (PMC13090138; doi:10.1002/sim.70529)

Severe acute respiratory illness

COVID-positive SARI

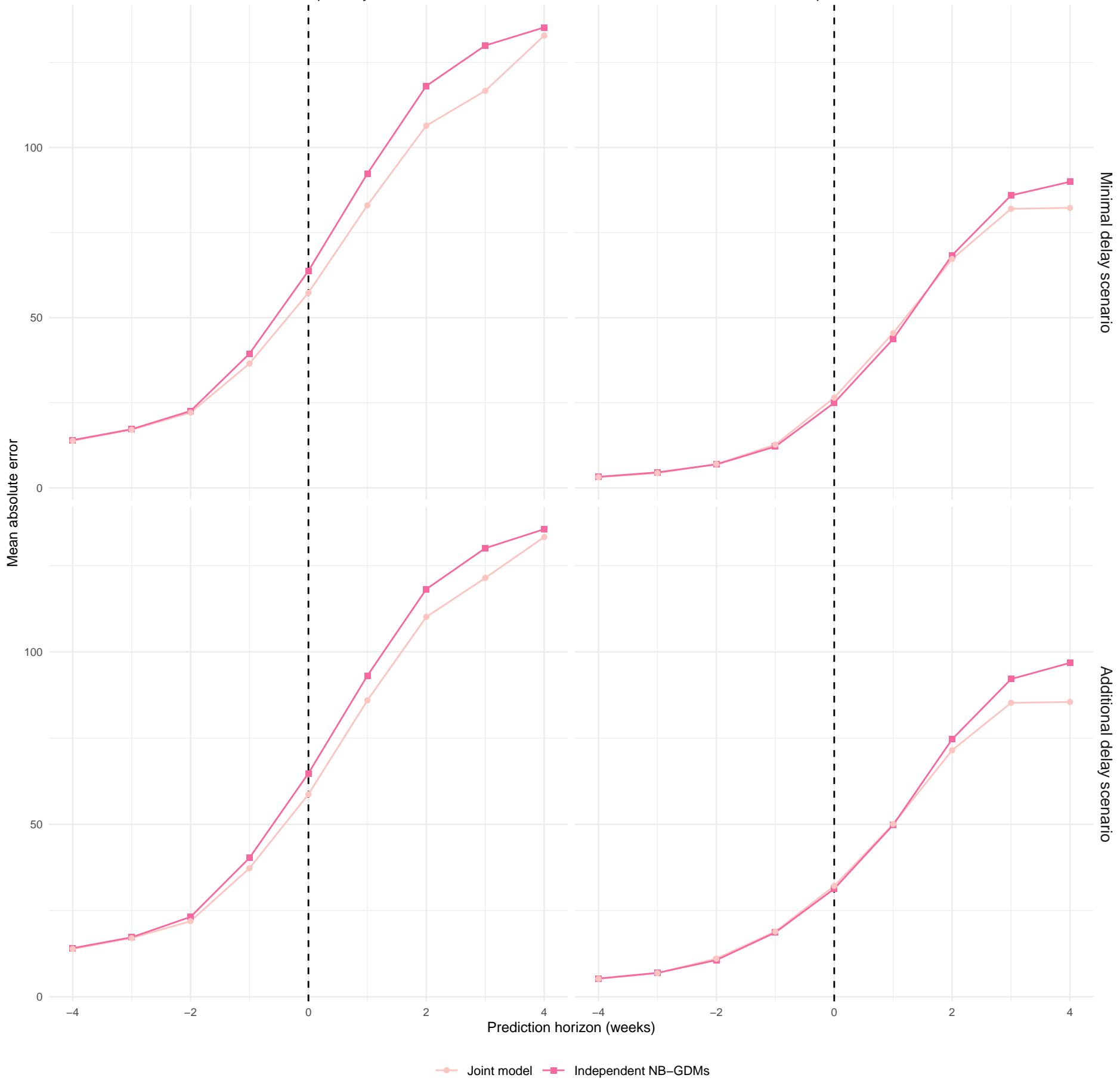

Supplement: Supplementary file 1 — Data S1. [file SIM-45-0-s001.zip › Software for joint Bayesian nowcasting/Plots/mae_plot_scenario.pdf]

MAE relative to NobBS

Minimal delay scenario

Additional delay scenario

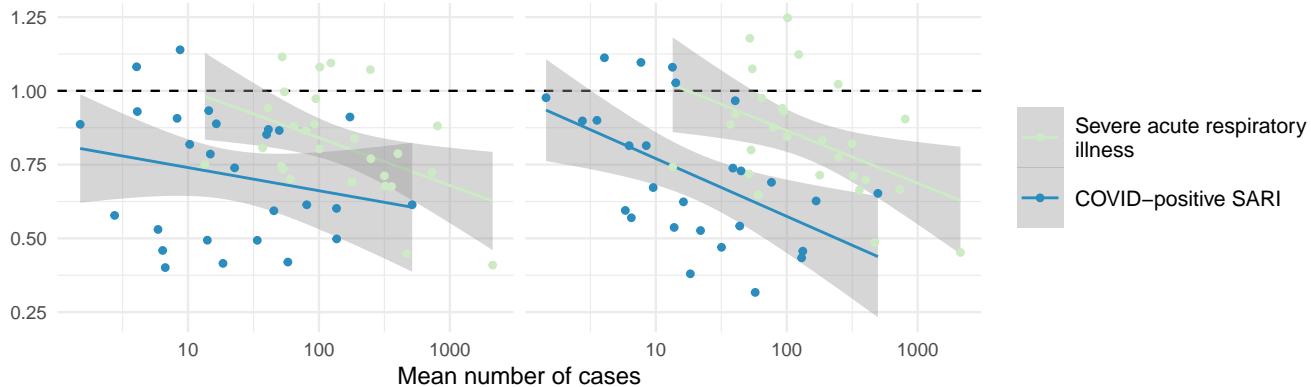

Supplement: Supplementary file 1 — Data S1. [file SIM-45-0-s001.zip › Software for joint Bayesian nowcasting/Plots/rel_mae.pdf]

Minimal delay scenario

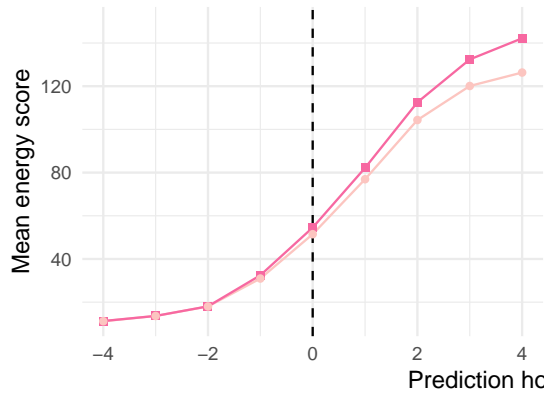

Additional delay scenario

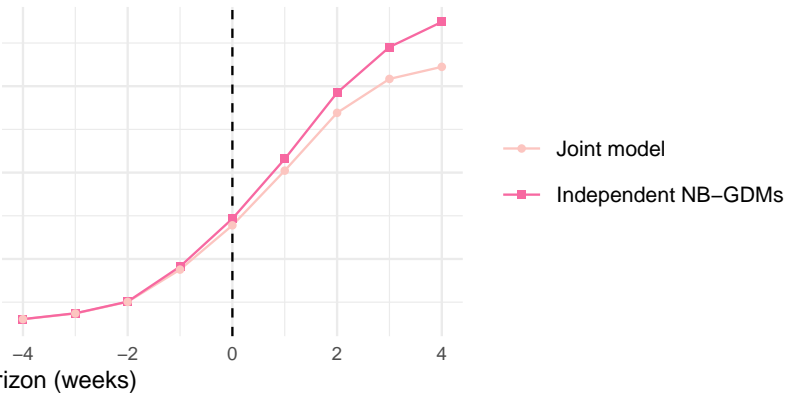

Supplement: Supplementary file 1 — Data S1. [file SIM-45-0-s001.zip › Software for joint Bayesian nowcasting/Plots/es_plot.pdf]

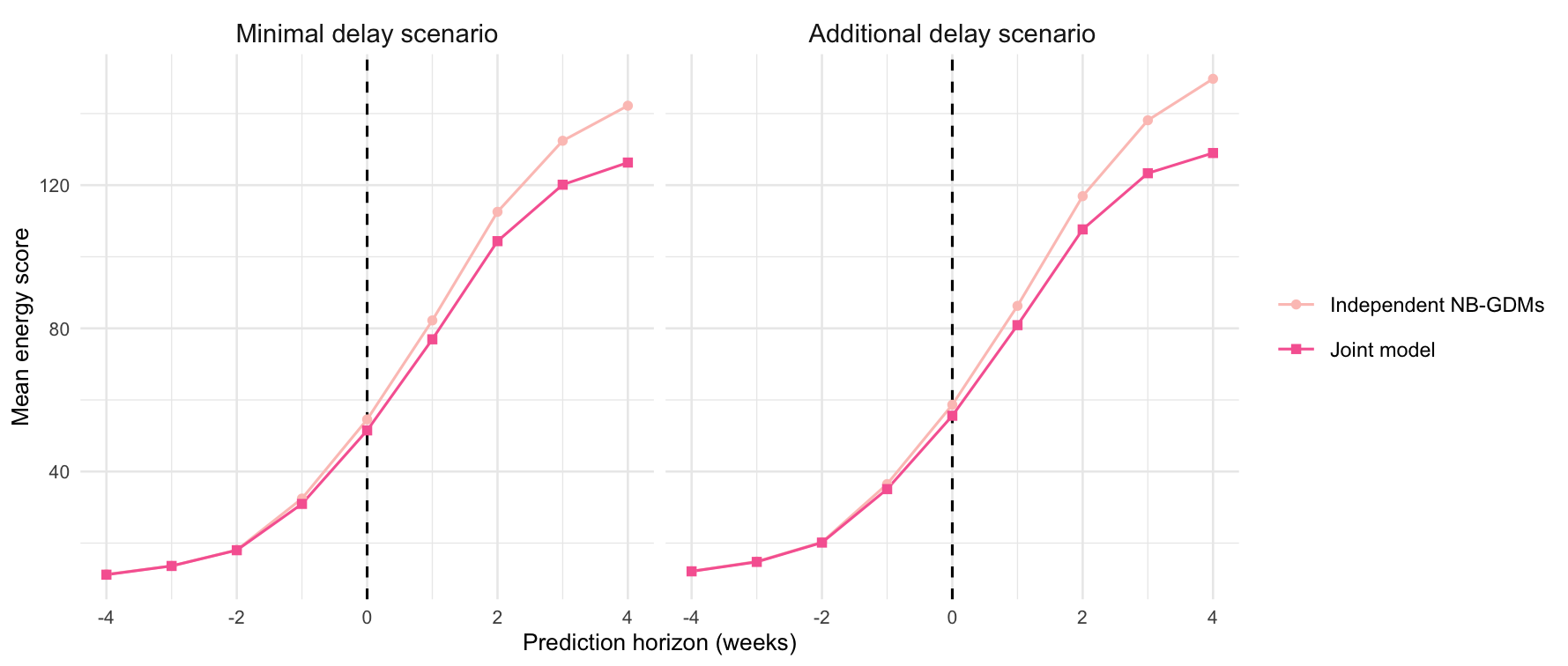

Supplement: Supplementary file 1 — Data S1. [file SIM-45-0-s001.zip › Software for joint Bayesian nowcasting/Plots/pres_plot_es.png]

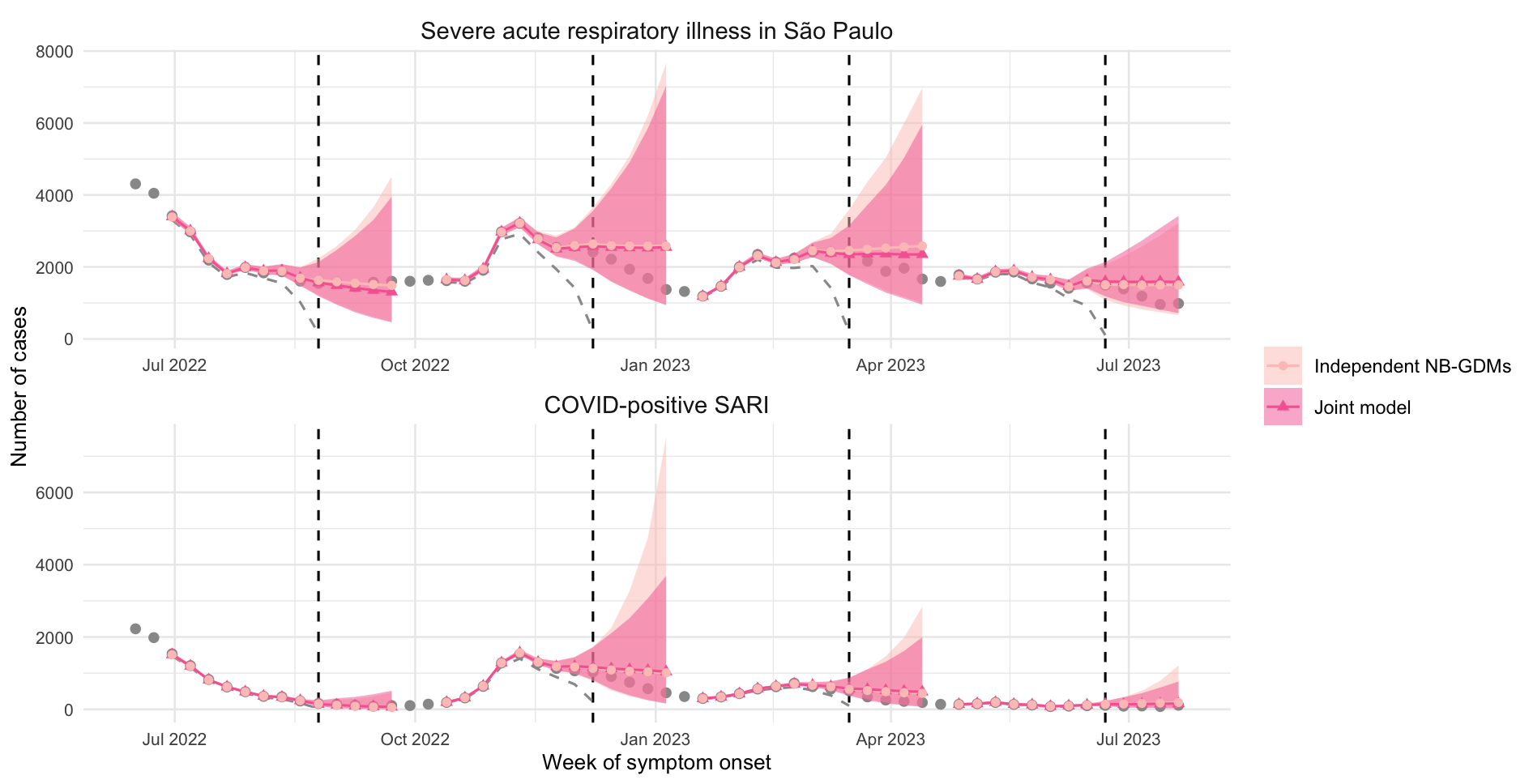

Supplement: Supplementary file 1 — Data S1. [file SIM-45-0-s001.zip › Software for joint Bayesian nowcasting/Plots/pres_plot_casts.png]

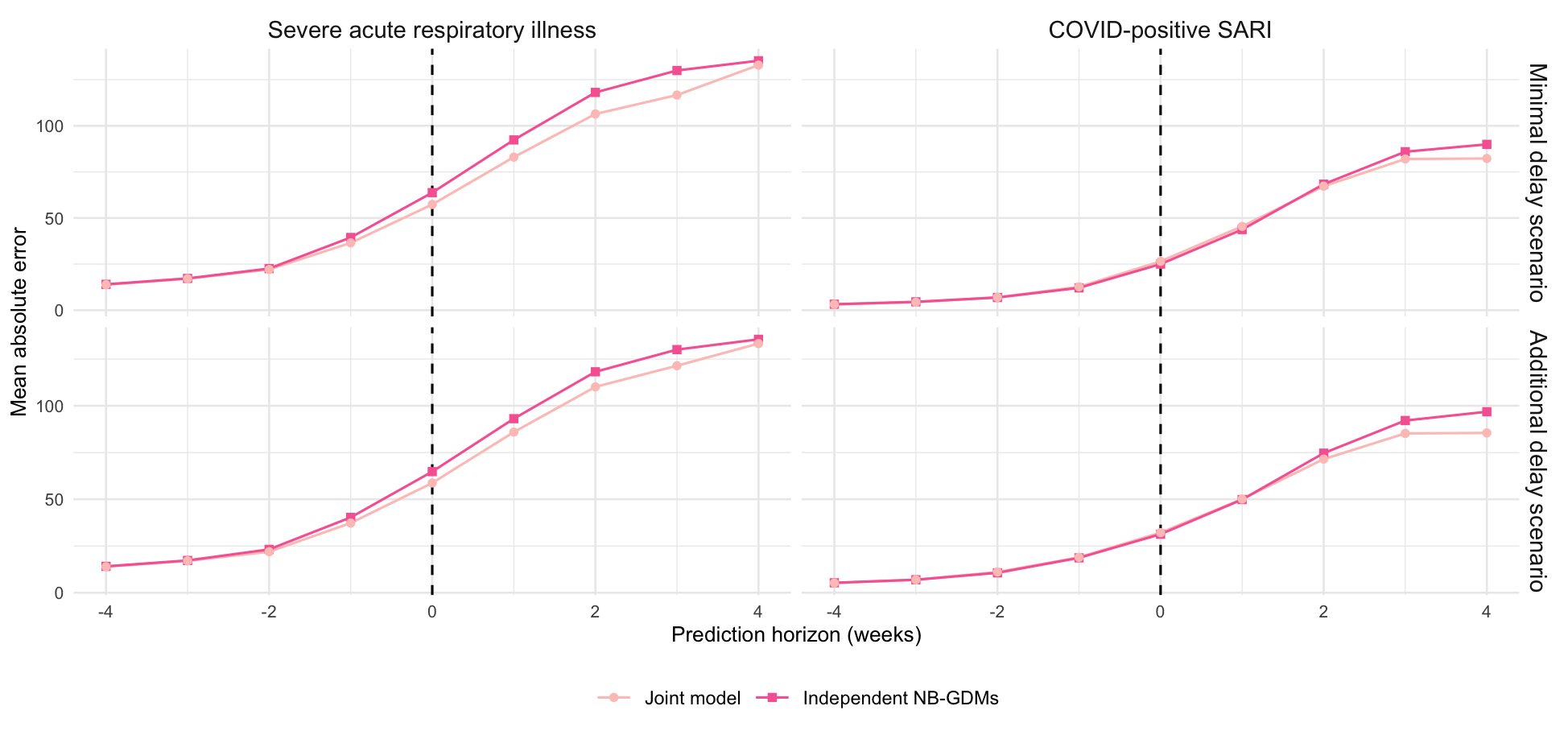

Supplement: Supplementary file 1 — Data S1. [file SIM-45-0-s001.zip › Software for joint Bayesian nowcasting/Plots/pres_plot_mae.png]

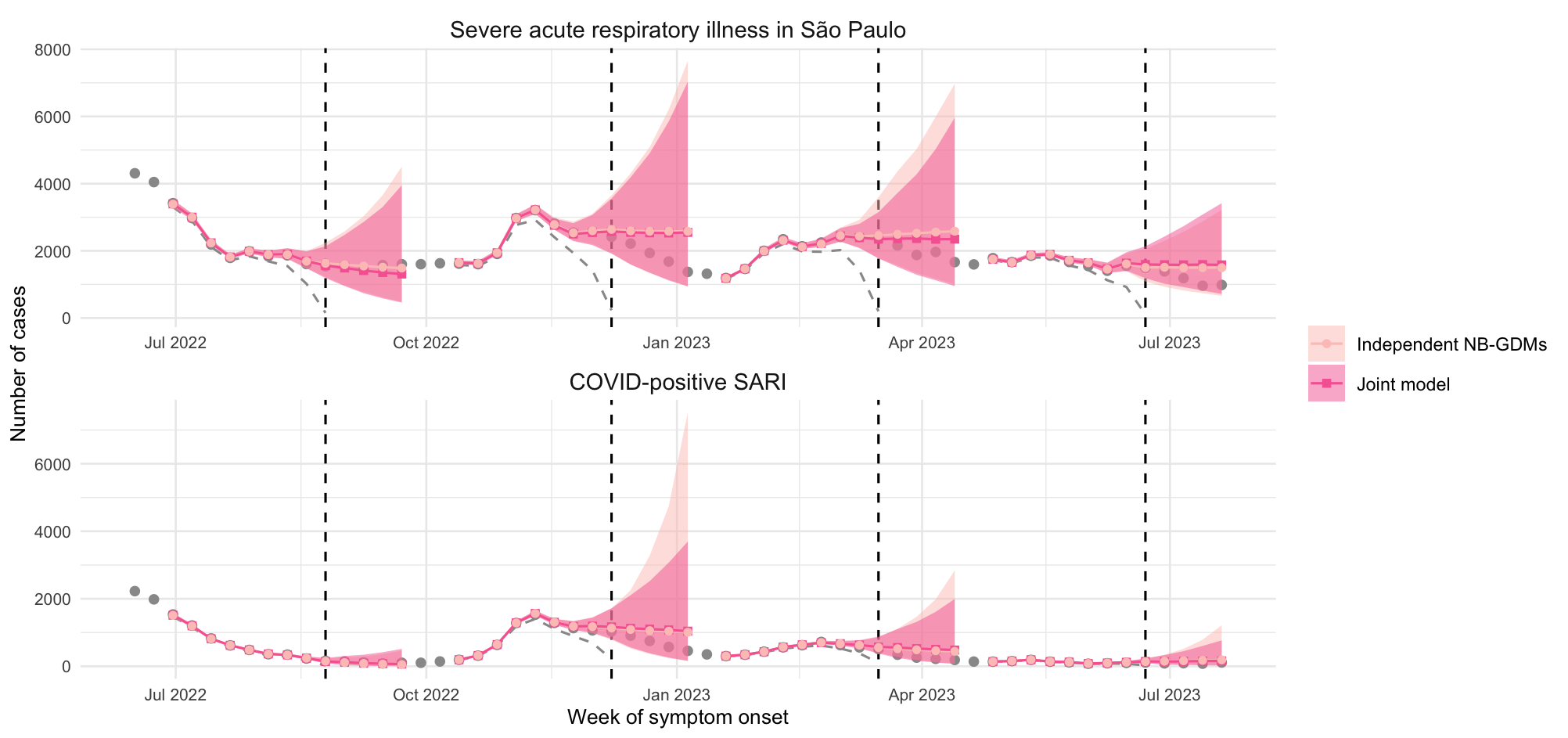

Supplement: Supplementary file 1 — Data S1. [file SIM-45-0-s001.zip › Software for joint Bayesian nowcasting/Plots/Rplot.png]

Severe acute respiratory illness

COVID-positive SARI

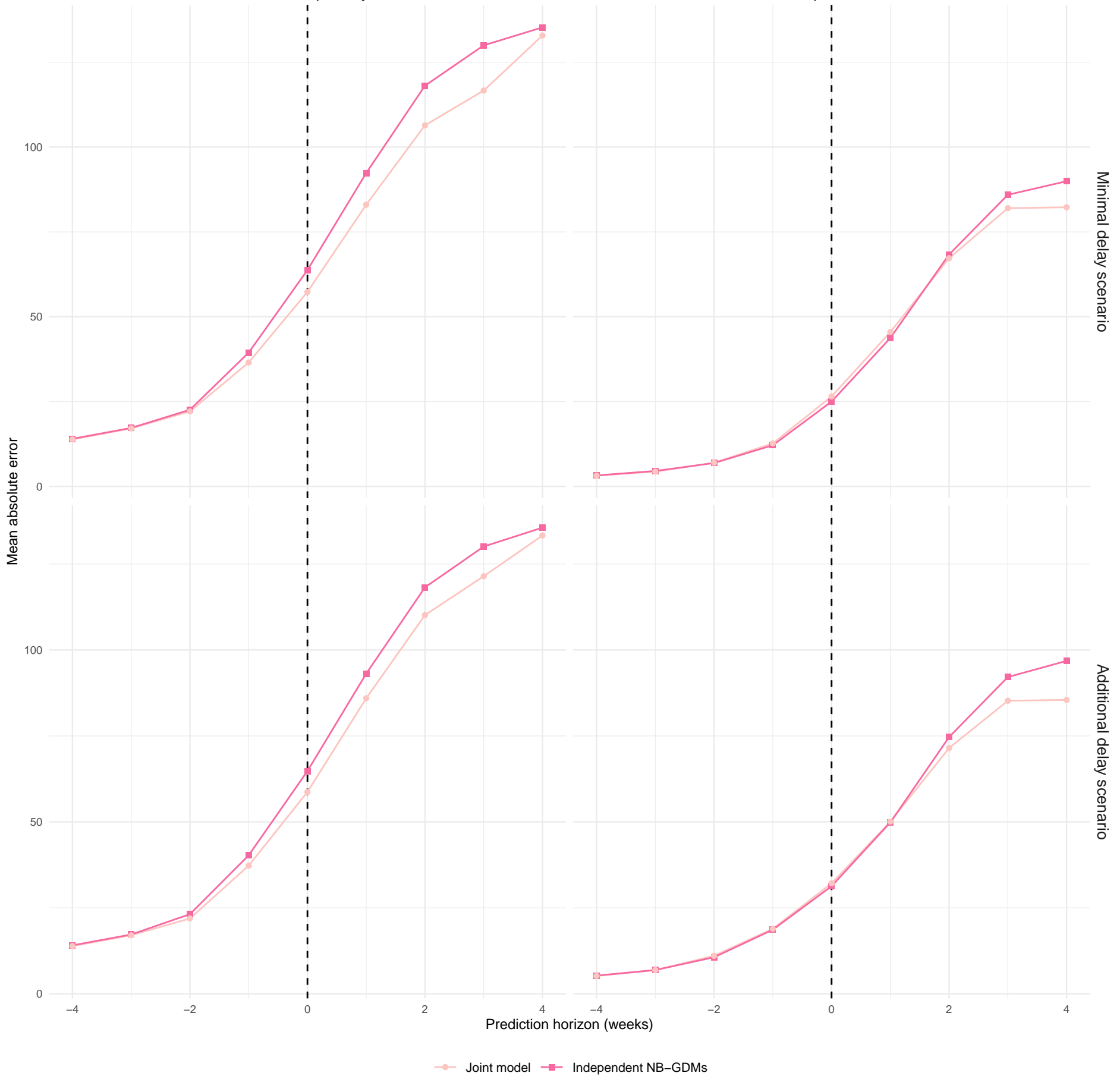

Supplement: Supplementary file 1 — Data S1. [file SIM-45-0-s001.zip › Software for joint Bayesian nowcasting/Plots/mae_plot_scen.pdf]

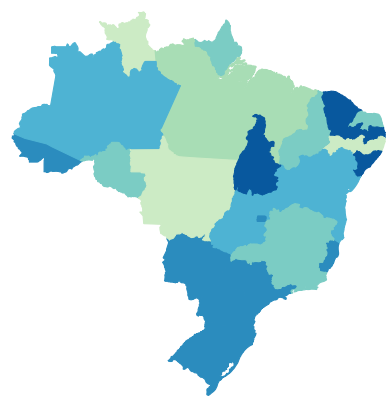

Median SARI reporting delay

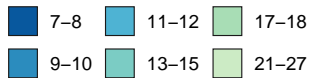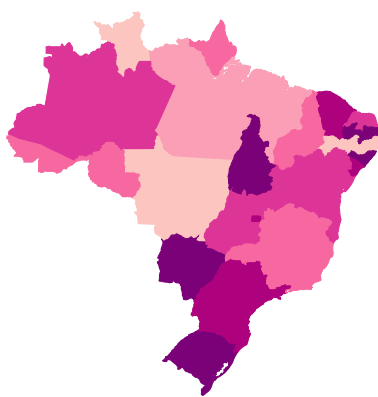

Interquartile range

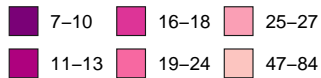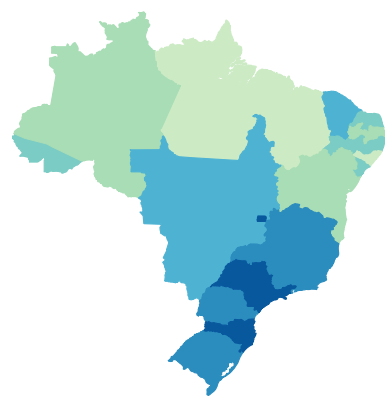

Human development index

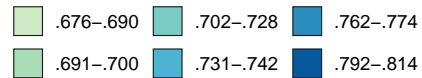

Supplement: Supplementary file 1 — Data S1. [file SIM-45-0-s001.zip › Software for joint Bayesian nowcasting/Plots/brazil_map.pdf]

Severe acute respiratory illness

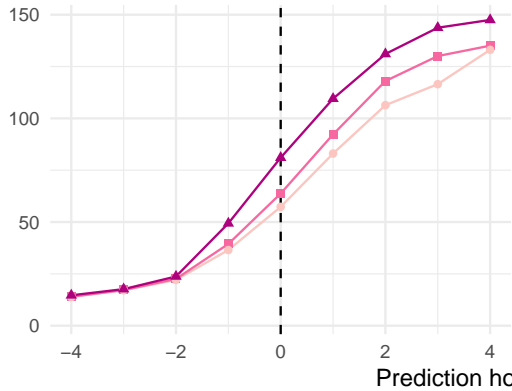

COVID-positive SARI

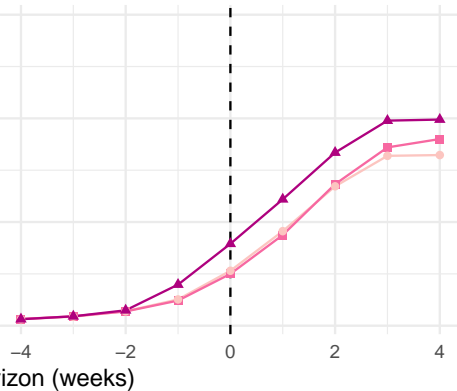

- Joint model
- Independent NB-GDMs
- NobBS

Supplement: Supplementary file 1 — Data S1. [file SIM-45-0-s001.zip › Software for joint Bayesian nowcasting/Plots/mae_plot.pdf]

## Severe acute respiratory illness in São Paulo

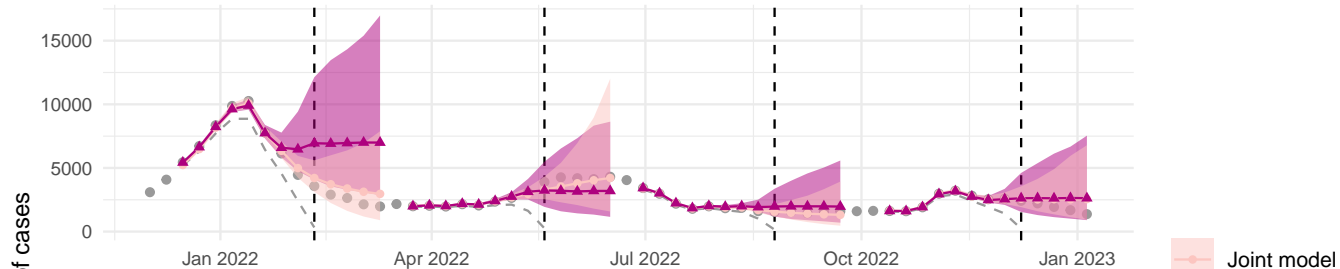

## COVID-positive SARI

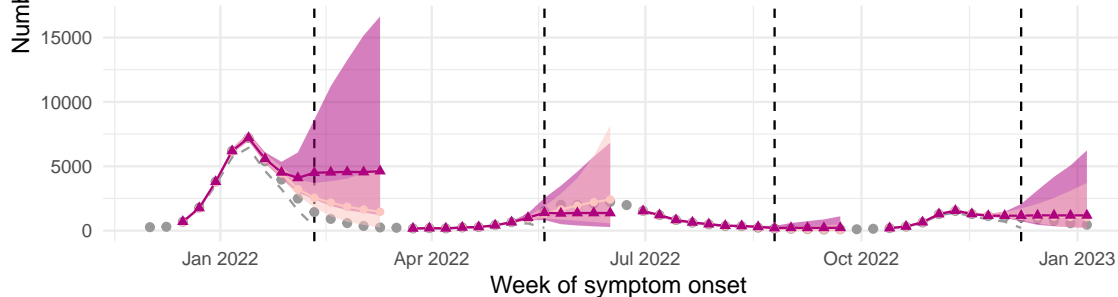

Supplement: Supplementary file 1 — Data S1. [file SIM-45-0-s001.zip › Software for joint Bayesian nowcasting/Plots/example_preds.pdf]

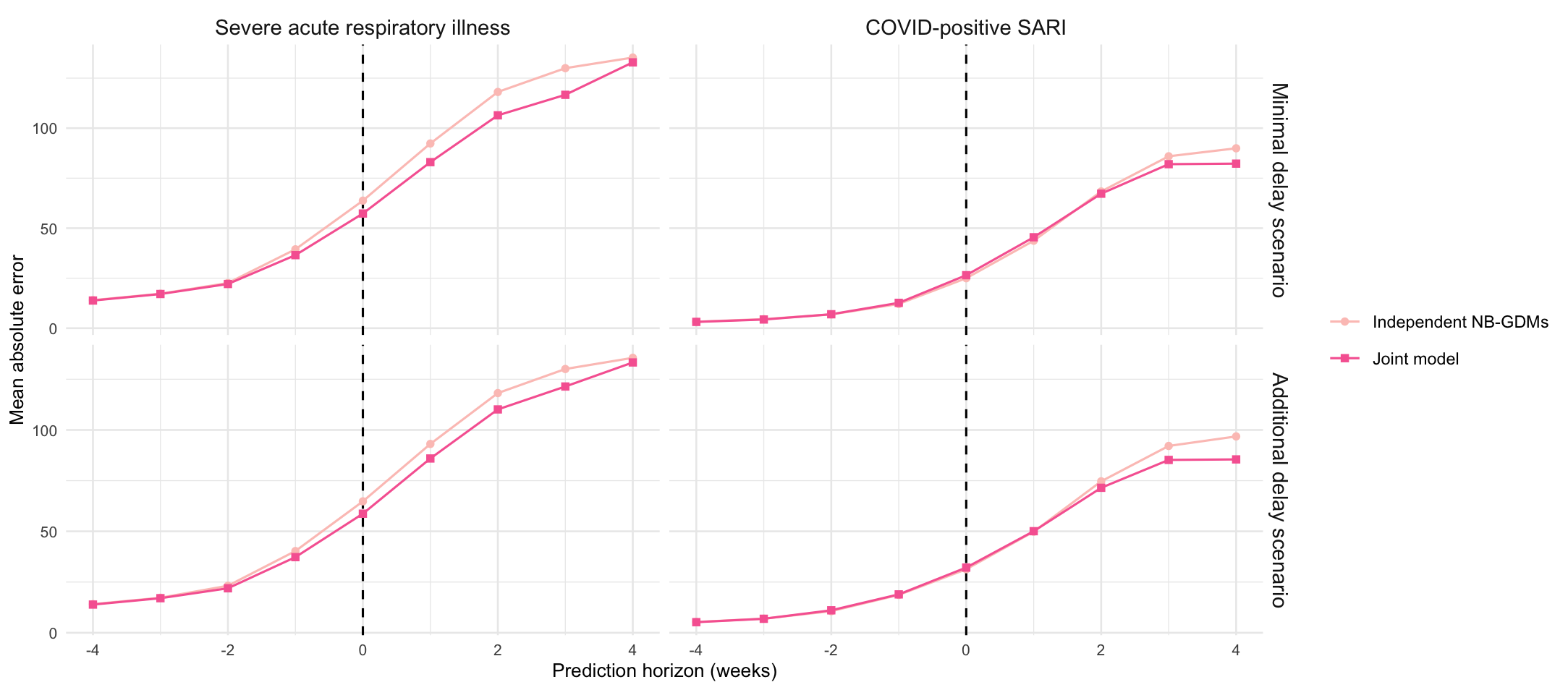

Supplement: Supplementary file 1 — Data S1. [file SIM-45-0-s001.zip › Software for joint Bayesian nowcasting/Plots/pre_plot_mae.png]
